# Chaplain development in Clinical Pastoral Education (CPE) in healthcare settings in England: A mixed methods study

**DOI:** 10.1371/journal.pone.0310085

**Published:** 2024-09-11

**Authors:** Csaba Szilagyi, Mark Newitt, Daniel Nuzum

**Affiliations:** 1 Rush University, Chicago, IL, United States of America; 2 Transforming Chaplaincy, Chicago, IL, United States of America; 3 KU Leuven, Leuven, Belgium; 4 Free Churches Group, London, United Kingdom; 5 Sheffield Teaching Hospitals NHS Foundation Trust, Sheffield, United Kingdom; 6 St Luke’s Hospice, Sheffield, United Kingdom; 7 University College Cork, Cork, Ireland; 8 Association of Clinical Pastoral Education, Ireland; Ulster University - Coleraine Campus: Ulster University, UNITED KINGDOM OF GREAT BRITAIN AND NORTHERN IRELAND

## Abstract

**Background:**

Clinical Pastoral Education (CPE) is the predominant specialised training for healthcare chaplains in several national contexts. CPE is spiritual care education that uses experiential and action-reflection learning methods to train diverse participants. However, CPE is not established for chaplaincy training in England. Currently, chaplaincy education in England lacks standardisation, leading to inequalities in entry into the profession and inconsistent training and career pathways. CPE has the potential to address these issues. We examined changes associated with participating in CPE and participants’ perceptions about their learning experience. We sought to evaluate the effectiveness of CPE as a viable chaplaincy education model in healthcare settings in England.

**Methods:**

Convergent mixed methods involved pre-post surveys and focus group sessions to examine the experiences and development of seven chaplains, with diverse experience levels and backgrounds, who participated in the pilot CPE unit in NHS England. We integrated thematic analysis and survey results.

**Results:**

We identified four overarching themes: Development pathways, Catalysts for development, Advantages of CPE for chaplaincy education, and Experiences with CPE course structure. Participants developed along various pathways: confidence, reflective practice, emotional intelligence, listening and attending skills, diversity in chaplaincy care, and spiritual assessment. Survey results confirmed several themes, indicating gains in chaplaincy capabilities, emotional intelligence, and counselling self-efficacy. Participants emphasised the advantages and effectiveness of the CPE model.

**Conclusions:**

Quantitative and qualitative findings converged to provide rich evidence that CPE generated personal and professional development, improving chaplaincy practice. General learning pathways moved from personal development, through the interpersonal learning context, and translated into chaplain competency. Participants endorsed CPE, as a robust and effective training model for chaplaincy in the English context, for those entering the profession and experienced chaplains alike. We conceptualised preliminary models for chaplain development and learning pathways in CPE that need validation and refinement by future research.

## Introduction

Clinical Pastoral Education (CPE) is the predominant specialised training for healthcare chaplains in several national contexts such as the USA, Canada, Ireland, Australia, and New Zealand and is often required for certification as a professional chaplain [1–7]. However, CPE is not established for chaplaincy training in healthcare settings in England.

Chaplaincy qualifications and education in England lack standardisation, leading to inequities in entry into the chaplaincy profession, particularly for diverse belief groups, and inconsistent training and career pathways. Highlighting these issues, the Caring for the Spirit [8] workforce strategy, concerned with enabling the chaplaincy workforce to make a greater contribution to healthcare in the National Health Service (NHS), identified key challenges facing the profession: broadening entry into chaplaincy especially in relation to diversity, expanding short and narrow career pathways, and developing specialised training for chaplaincy. However, the strategy was never fully implemented and uneven models for chaplaincy education and professional development remained.

As an internationally established and recognised training programme [1–7], CPE addresses the core issues of standardised training, equitable access, diversity, and professional development. CPE is spiritual care education that uses experiential and action-reflection-action learning methods to train participants of diverse religious, spiritual, and philosophical backgrounds [9]. These students learn from clinical care experiences with the diverse individuals they serve, and from critical reflection, feedback, and interactions within their peer group. Each group is led by a certified CPE supervisor/educator. One CPE unit entails 400 hours of training. Primary educational components include supervised clinical practice, peer group process, individual clinical supervision, verbatim case discussions, and didactic instruction [7, 9]. CPE adheres to established standards and learning objectives set forth by accrediting agencies, such as the ACPE: The Standard for Spiritual Care & Education [10] in the USA, the Canadian Association for Spiritual Care / Association canadienne de soins spirituels (CASC/ACSS) [11], the Australia and New Zealand Association for Clinical Pastoral Education Ltd (ANZACPE) [12], and the Association of Clinical Pastoral Education (Ireland) Ltd (ACPEI) [13]. Generally, CPE objectives aim to integrate personal, pastoral/spiritual, and professional growth and develop capabilities for spiritual care practice with diverse clients [10, 13].

To date, research on CPE has paid little attention to measurable outcomes and changes associated with CPE [14–16]. However, the limited previous research generally showed positive effects of CPE on personal, professional, pastoral, and competency development for spiritual care [17–21], and most students valued the distinct learning methods of CPE [21, 22]. Additionally, research on the relationship between CPE learning outcomes and professional chaplain competencies is scarce [14, 15].

This study aimed to examine the changes associated with participating in a CPE unit and the perceptions of the participants about their CPE learning experience. Furthermore, we sought to evaluate the effectiveness of CPE as a viable chaplaincy education model in England by investigating the outcomes of a pilot CPE unit in NHS England. This evaluation study was commissioned as part of a Clinical Pastoral Education (CPE) pilot project funded by Health Education England through 2022–2023.

## Methods

**Fig 1:**
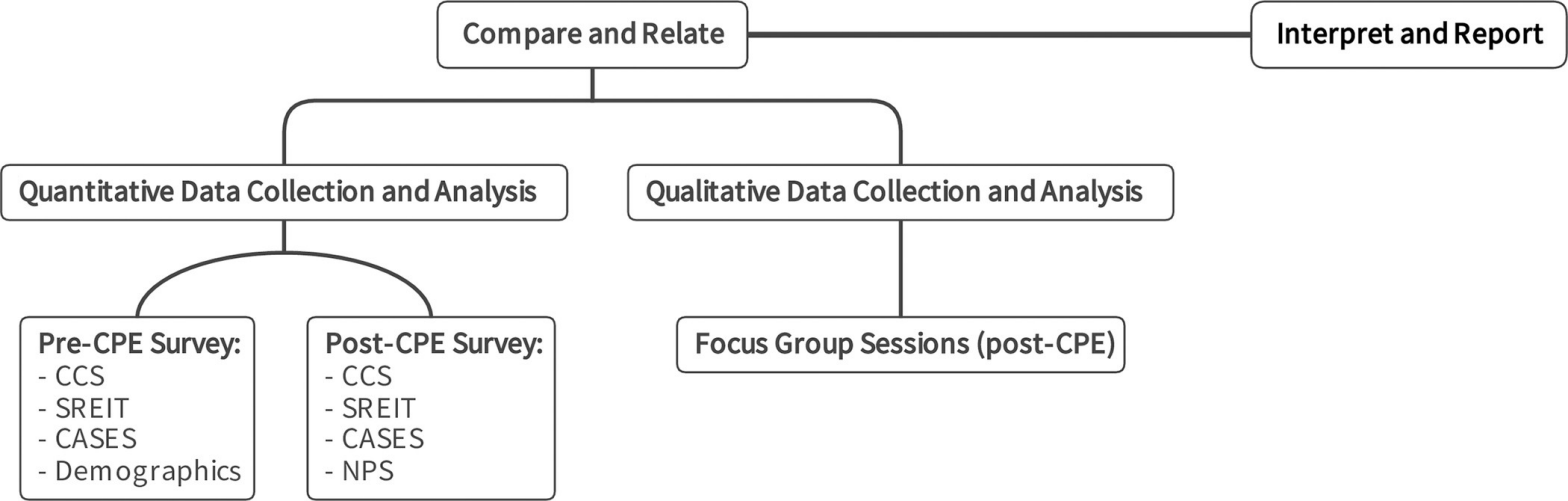
Mixed methods design and data collection. Fig 1 presents the convergent mixed methods research design, including data collection points and survey instruments. Survey instruments: CCS: Chaplain Capabilities Scale; SREIT: Self-Report Emotional Intelligence Test (SREIT) [26, 27]; CASES: Counselor Activity Self-Efficacy Scales (CASES) (Parts 1 and 2) [28]; NPS: Net Promoter Score. CPE: Clinical Pastoral Education.

This mixed methods study employed a convergent quantitative-qualitative design in two stages (Fig 1). First, we administered quantitative online surveys at the beginning and end of the CPE unit to investigate changes resulting from CPE. Second, we conducted two focus group sessions to further explore perspectives on the learning, changes, and course structure that participants experienced in CPE. Qualitative and quantitative data were initially analysed separately and then integrated during further data analysis, interpretation, and reporting [23]. We integrated qualitative and quantitative findings through narrative, weaving them together theme-by-theme and giving priority to qualitative findings [23]. Using mixed methods enabled us to confirm qualitative and expand quantitative findings given the small sample size. We used the Good Reporting of a Mixed Methods Study (GRAMMS) [24] and the Standards for Reporting Qualitative Research (SRQR) [25] protocols to guide the reporting (see S1 and S2 Tables for checklists). Additional information regarding the ethical, cultural, and scientific considerations specific to inclusivity in global research is included in the (S1 Checklist).

### Participants and setting

For a purposive sample, we recruited eligible participants who were chaplains enrolled in the pilot CPE unit in NHS England from October 2022 to February 2023. The NHS Health Research Authority (HRA) decision tool was utilised to evaluate if review by an NHS Research Ethics Committee (REC) was required [29]. It indicated that an NHS REC review was not required. This is consistent with the statement in the accompanying algorithm that REC review is not required for NHS or social care staff recruited as study participants due to their professional roles [30]. The study incorporated multiple points of informed consent at pre and post surveys and focus group sessions. At each, participants were informed about the research objectives and procedures, efforts to protect their privacy and confidentiality, how the data will be used and protected, and their voluntary participation or refusal to participate will not have any consequences. They were assured that their decisions to participate and their responses will not have any bearing on their educational or professional evaluations. At the start of the surveys, participants consented online in writing. Their consent was also taken verbally (witnessed by moderators CS and MN) at the beginning of the focus group sessions. Data was collected and managed securely to maintain confidentiality and privacy.

### The Clinical Pastoral Education (CPE) unit

The educational intervention involved a unit of CPE that was run as a hybrid programme according to the standards and learning outcomes of the Association of Clinical Pastoral Education (Ireland) Ltd [13]. The programme lasted fourteen weeks based at Sheffield Teaching Hospitals. The unit consisted of a total of 400 hours of training, which included 180 didactic hours, with 40 hours of interpersonal group process (IPG), in addition to 220 hours of supervised practice in spiritual care. The group met in person for twelve days during the unit. The remaining didactic and interpersonal group days were conducted using synchronous video technology (Microsoft Teams). The programme was developed based on ACPEI standards and the group’s learning needs. The group was supervised by a certified ACPEI supervisor/educator. The didactic presentations were drawn mainly from healthcare chaplaincy experts from the UK context. All participants successfully completed the unit.

### Quantitative methods: Pre-post surveys

We invited potential participants by emailing the survey information and link. We collected baseline survey data October 17–28, 2022, and post-CPE data February 7–17, 2023, using a Microsoft Forms electronic data capture tool. Participants completed the following repeated survey instruments in addition to a demographic questionnaire (pre-CPE) and a closed and an open-ended Net Promoter Score (NPS) ("likelihood to recommend") items (post-CPE). Fig 1 shows survey instruments and data collection points.

#### Chaplain capabilities

The authors developed a 14-item scale informed by the Spiritual and Religious Care Capabilities and Competences for Healthcare Chaplains framework by the UK Board of Healthcare Chaplaincy [31] and the ACPEI CPE Learning Outcomes [13] to assess how participants’ chaplaincy competences changed as a result of CPE. To date, this scale has not been validated. Respondents indicated on a 5-point scale, from 1 (not at all) to 5 (extremely), how capable they felt of effectively using each spiritual care skill with most individuals over the next week. Average scores were calculated for each item and the entire scale.

#### Emotional intelligence (EI)

The Self-Report Emotional Intelligence Test (SREIT) was used to measure respondents’ characteristic adaptive emotional functioning [26, 27]. This 33-item instrument, based on the Salovey and Mayer EI model (1990), assessed how individuals characteristically appraise emotions in themselves and others, express emotions, regulate emotions in themselves and others, and utilise emotions. Respondents rated the extent to which each statement described them on a 5-point scale from 1 (strongly disagree) to 5 (strongly agree). The total score was the sum of all items after reverse coding three items, with higher scores indicating greater emotional intelligence (ranging 33–165). We also calculated subscale and overall results by using average scores to allow for comparing scales with varying numbers of items [27].

#### Counselling self-efficacy

To measure counselling self-efficacy, we used Part 1 and Part 2 of the Counselor Activity Self-Efficacy Scales (CASES) [28]. CASES assessed participants’ beliefs about their ability to perform various helping and counselling skills. Respondents reported their level of confidence in their ability to use helping and counselling skills effectively on a 10-point scale from 0 (no confidence) to 9 (complete confidence). Average scores were calculated for each CASES scale assessing Helping Skills Self-Efficacy, including Insight Skills, Exploration Skills, and Action Skills, and Session Management Self-Efficacy.

#### Statistical analysis

Descriptive statistics were generated to describe the participant characteristics and survey responses using Microsoft Excel and JASP version 0.18.3. Categorical data are presented as frequencies (percentages) and continuous data as medians (interquartile ranges [IQR]) with ranges. Instruments were scored as described under each scale. These statistics were also used to generate the respective tables and figures. Due to the small sample size, there was no effort to draw quantitative inferences and test whether changes in scores on the scales were statistically significant.

### Qualitative methods

Participants were invited by email and provided the focus group information beforehand. Two 90-minute focus group sessions were held via Zoom on February 21 and March 2, 2023, after the post-CPE survey was completed. The sessions followed an interactive focus group approach using a semi-structured interview guide. CS and MN served as moderators. DN, the CPE supervisor/educator, did not attend the focus groups. Questions explored participants’ perspectives on (a) their learning and development in CPE, (b) how CPE impacted their capabilities and identity as chaplains, and (c) the utility of CPE for chaplaincy training. Focus group questions are available in S3 Table.

#### Qualitative analysis

We performed reflexive thematic analysis as an inductive approach to examine patterns of meaning related to our research questions in focus group data [32, 33]. The focus group sessions were recorded and transcribed. The first author (CS) performed the analysis and reflexively considered his location related to the participants and the research topic. He is an ACPE Certified Educator, chaplain, and researcher with graduate degrees in Divinity, Education, and Management, and is a European male living in the USA. He did not supervise this group of CPE students and had no interactions with them except for the focus group sessions. He familiarised himself with the data, inductively coded the transcripts using ATLAS.ti Version: 23 qualitative data analysis software, and generated initial themes. He defined themes and developed a thematic frame regarding participants’ perceptions. Although a single coder is typical in thematic analysis [34], the other two authors (MN and DN) reviewed the data and the development of themes to ensure interpretive depth, internal coherence, and meaningful reflection of participant views regarding the research questions. DN served as the CPE supervisor/educator for the participants, and MN acted as the main link between ACPEI and NHS for the CPE pilot.

## Results

### Participant characteristics

All seven chaplains undertaking CPE completed the pre-post surveys and participated in the focus group sessions, yielding a 100% response rate. The median participant age was 53 (IQR = 6.5) years, and the majority was female (85.7%). The composition was reasonably diverse in terms of ethnic groups and religious/spiritual backgrounds represented, considering the small CPE group size. Respondents’ years of chaplaincy experience ranged from 1 to 18 years, with the median length of experience being 5 (IQR = 9.3) years. Table 1 reports additional participant characteristics.

**Table 1:** Participant characteristics.

Characteristic (n = 7)	
Age, median (IQR) range	53 (6.5); 41–68
Gender, N (%)	
Female	6 (85.7)
Male	1 (14.3)
Non-binary	0 (0)
Ethnic Group, N (%) ^a^	
Asian	2 (28.6)
Black	0 (0)
Chinese	0 (0)
Mixed	0 (0)
White	5 (71.4)
Other	0 (0)
Faith or Belief Background ^b^	
Catholic	2 (28.6)
Church of England	2 (28.6)
Christian Faith Church	1 (14.3)
Islam	1 (14.3)
Non-Religious	1 (14.3)
Years of Chaplaincy Experience, median (IQR) range	5 (9.3); 1–18
Current Level of Working as a Chaplain, N (%) ^b^	
Band 6	5 (71.4)
Band 7	1 (14.3)
Band not reported	1 (14.3)
Full time	2 (28.6)
Part time ^c^	4 (57.1)
Full or part time not reported	1 (14.3)
Contexts of Chaplaincy, N (%) ^b^^,^ ^d^	
General Acute	4 (57.1)
Mental Health	2 (28.6)
Paediatric	2 (28.6)
Palliative Care	2 (28.6)
Accident & Emergency Department	1 (14.3)
Community	1 (14.3)
Primary Care—General Practice	1 (14.3)
Years of Prior Non-Chaplaincy Pastoral Experience, median (IQR) range	17 (13); 8–40

^a^ NHS workforce broad ethnic group categories were used.

^b^ Participants self-described the characteristics in free-text responses.

^c^ Part time meant working up to 30 hours per week as a chaplain.

^d^ Participants indicated more than one context per person.

### Themes of chaplain development in Clinical Pastoral Education (CPE)

**Fig 2:**
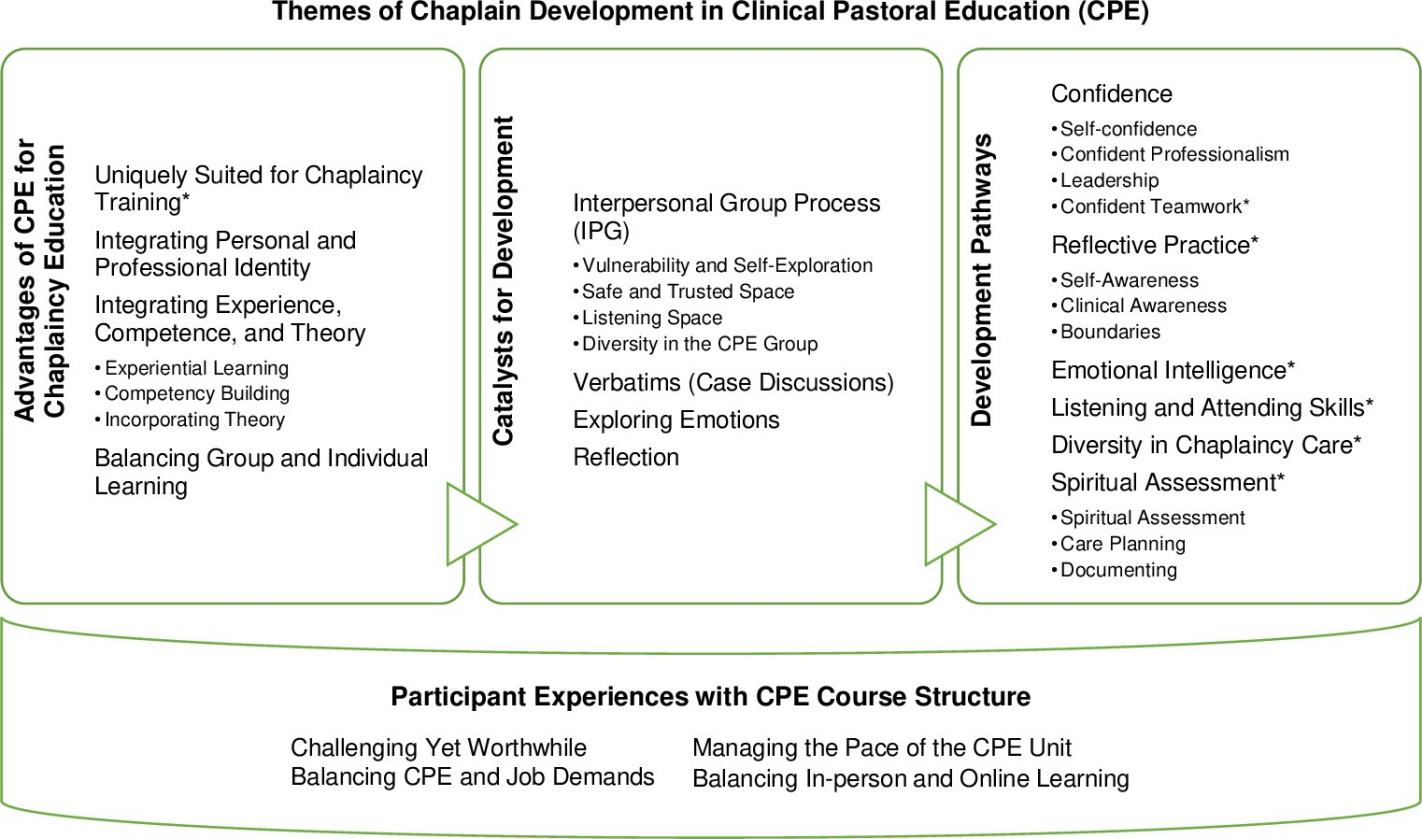
Themes of chaplain development in Clinical Pastoral Education (CPE). Fig 2 depicts the chaplain Development Pathways in Clinical Pastoral Education (CPE) that were fostered by the Catalysts for Development and the distinct features reflecting the Advantages of CPE for Chaplaincy Education and shaped by Participant Experiences with CPE Course Structure. Asterisks (*) denote qualitative themes confirmed by quantitative survey data.

Our analysis generated four overarching themes: (1) Development Pathways, (2) Catalysts for Development, (3) Advantages of CPE for Chaplaincy Education, and (4) Participant Experiences with CPE Course Structure. Fig 2 notes the intersection of these themes and their roles in chaplain development toward specific outcomes. On the right, the pathways that the participants identified for their development in CPE are shown, reflecting clear forward motion and gains from before to after CPE. Displayed from left to right are the advantages of the CPE model for chaplaincy education and the catalysts for development, which involve instrumental factors that participants highlighted as fostering their growth in key areas. Finally, participants commented on challenging and worthwhile aspects of their experiences with the structure of the CPE course, whose underlying dynamics shaped the learning environment (shown beneath the developmental facets). Additionally, quantitative pre-post survey results confirmed several qualitative themes (denoted with asterisks), indicating gains in chaplaincy capabilities, emotional intelligence, and counselling self-efficacy (S4 and S5 Tables and S1 Fig provide a summary of survey results).

### 1. Development pathways in CPE

**Fig 3:**
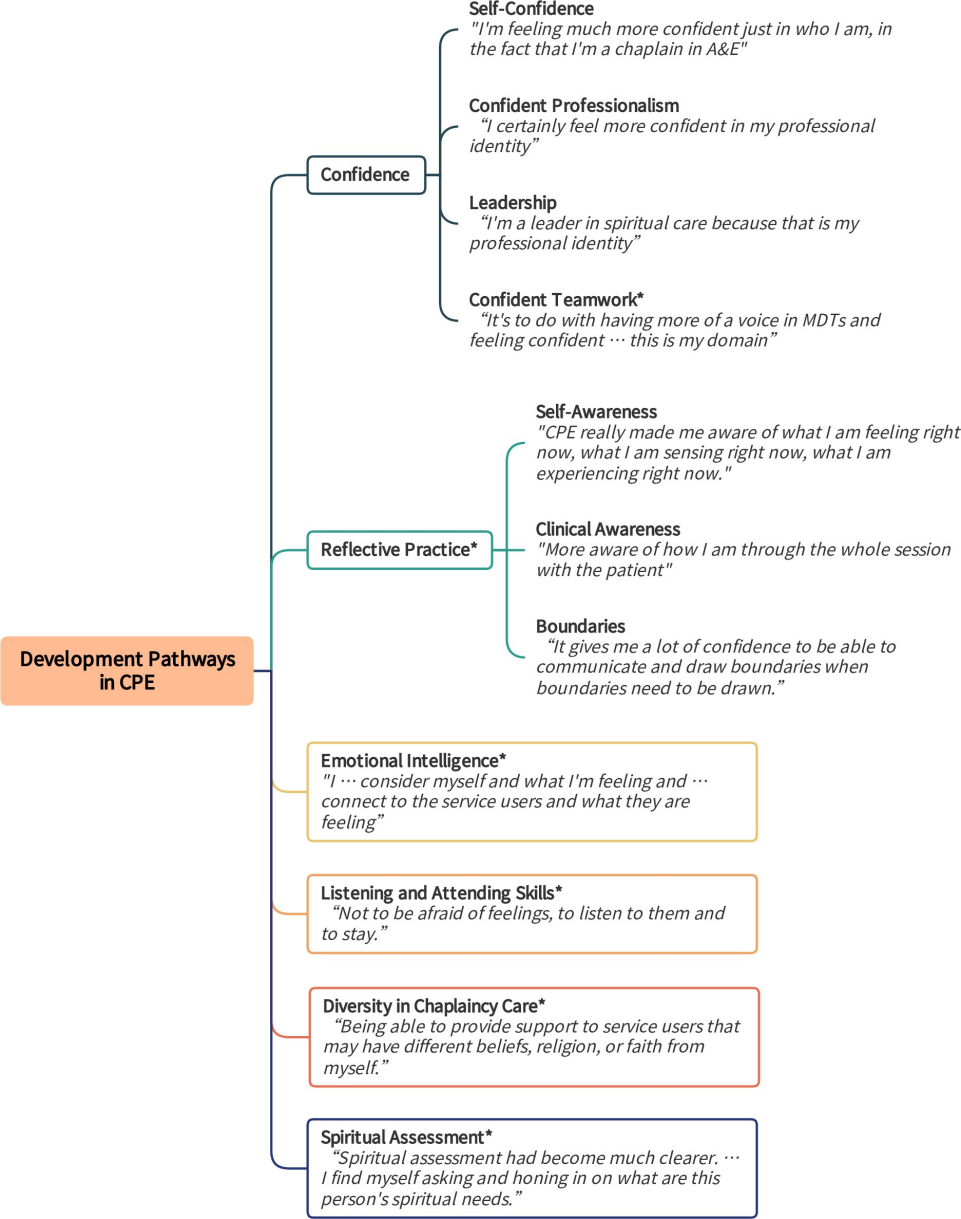
Development pathways in Clinical Pastoral Education (CPE). Fig 3 provides themes and subthemes with exemplary quotes regarding Development Pathways in Clinical Pastoral Education (CPE). Asterisks (*) denote qualitative themes confirmed by quantitative survey data.

Participants described several pathways for their development in CPE. They clearly grew as a result of CPE, as seen in the progress in these themes (Fig 3). These gains reflected the participants’ personal and professional development and integration. Each of these themes will be examined in turn.

#### Confidence

**Fig 4:**
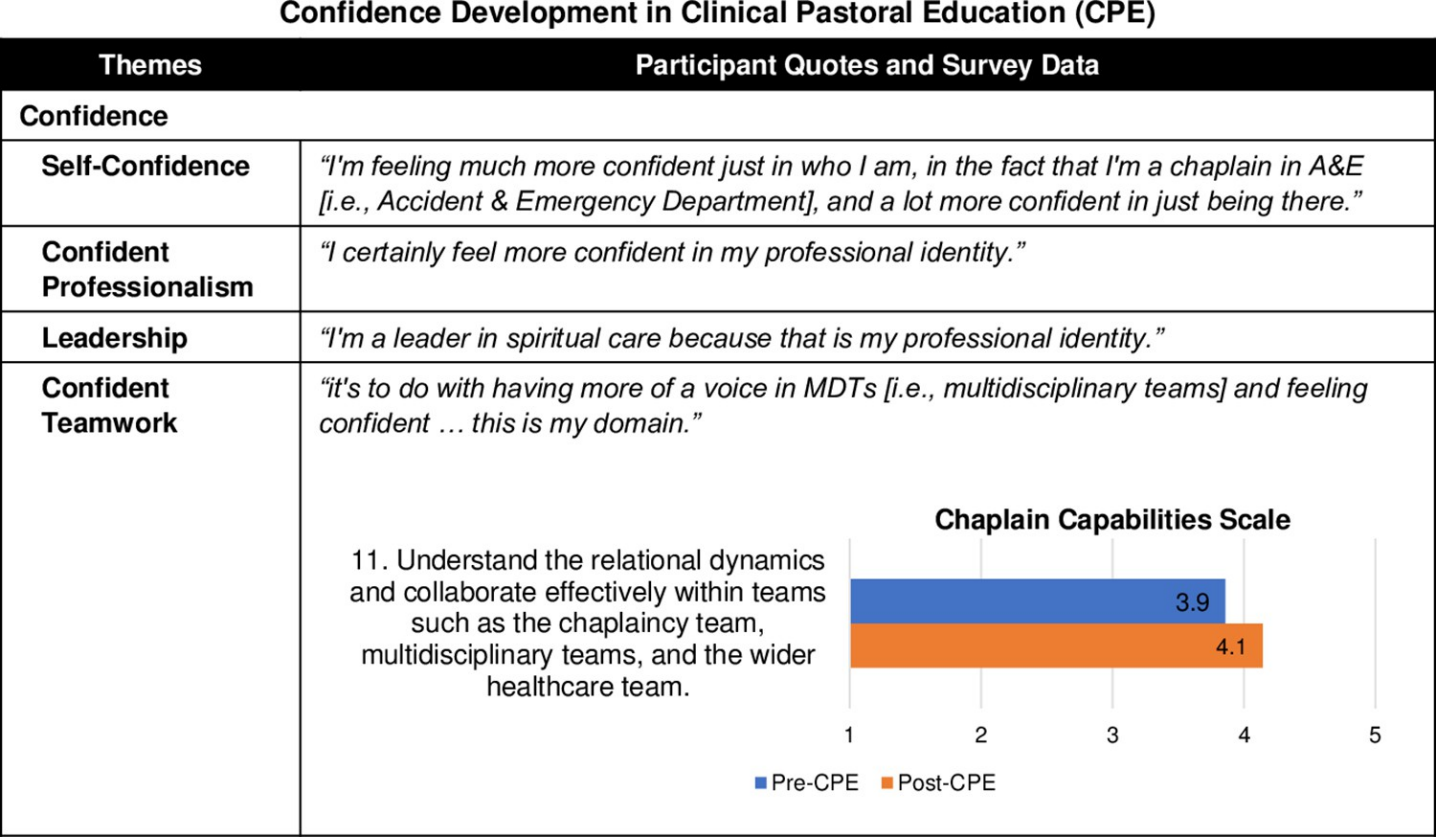
Confidence development in Clinical Pastoral Education (CPE). Fig 4 provides a joint display comparing qualitative themes with exemplary quotes and survey results with Chaplain Capabilities Scale mean scores. The 14-item CCS was developed by the authors and was informed by the Spiritual and Religious Care Capabilities and Competences for Healthcare Chaplains by the UK Board of Healthcare Chaplaincy [31] and the ACPEI CPE Learning Outcomes [13].

Participants described gaining confidence during CPE across four domains: personal, professional, leadership, and teamwork (Fig 4). Developing confidence in these arenas was one of the most consistent themes.

*Self-Confidence*. Participants reflected on cultivating self-confidence: *"I’m feeling much more confident just in who I am*, *in the fact that I’m a chaplain in A&E [i*.*e*., *Accident & Emergency Department]*, *and a lot more confident in just being there*.*"* It involved increasing their ability to trust themselves more fully. One participant noted, *"for me*, *it was trusting my own intuition*. *I would do that much more now*.*"* They felt more poised to express their authentic self and bring it into interactions with others as chaplains. CPE equipped them *"to be able to take 100% of me to whomever else I am seeing*.*"* Another interviewee highlighted, *"I just sense that I would be able to voice or express myself*, *where previously I might not have felt so confident in doing that*.*"*

*Confident Professionalism*. Participants highlighted significant ways in which they gained confidence in their professionalism and identities as healthcare chaplains. A participant offered an example of asserting their voice on the multidisciplinary team (MDT) from a stance of self-assured professionalism:

"*I certainly feel more confident in my professional identity when I am talking to the members of the MDT. … pre-CPE, … I would have felt not so confident. I am sure about how valuable my role could be. … because CPE has helped me to really deal with this difficult situation with a lot of professionalism. I don’t think I would have said what I said if I didn’t have this confidence that I get from the CPE."*

Participants gained a deeper appreciation of their role’s value to patient care and the team:

"*I feel I undervalued myself in comparison to the other members of the multidisciplinary team. … But with the CPE, … it changes how I value myself and how I value the chaplaincy work we do. We really are doing very hard work, and it’s because we are professional, we are able to do it."*

*Leadership*. Building on self-assurance and professionalism, participants more readily took leadership actions in their chaplain roles and collaborations with colleagues. They recognised that leadership is integral to chaplains’ professional identity and practice, noting: *"I’m a leader in spiritual care because that is my professional identity*.*"* Participants described leadership stemming from being the spiritual care specialist within the team: *"it was a realisation of that in CPE that I do have a leadership role*, *because I’m leading in spiritual care in that environment*. *… I have a leadership role in owning that*.*"*

Participants also described how they successfully exercised leadership with fellow chaplains and colleagues. An interviewee said, *"I am more confident*, *with … the chaplaincy team*, *so I would be able to participate more freely and take leadership within that team when it was necessary*.*"* Another commented, *"I think pre-CPE*, *I would have just been assisting my colleague in this staff support*, *but now I felt that we were co-leading it and that I had just as much stuff to bring as my colleague*.*"*

*Confident Teamwork*. Participants learnt to exercise more confidence, professionalism, and leadership in working with multidisciplinary and chaplaincy team members: *"it’s to do with having more of a voice in MDTs and feeling confident … this is my domain*.*"* CPE directly enhanced their capacity for teamwork with multidisciplinary teams (MDT), as a participant called it, *"claiming my space in the MDT*.*"* They developed more of an equal standing when communicating and collaborating with colleagues. *“[CPE] gave me more confidence in the role of chaplain*.*”* Recalling a debriefing session for staff, the participant continued:

“*Maybe before, I would have just listened to the consultant facilitate that debriefing session, but I think I intervened a lot more than I would have done, with questions, or with the nurses who are struggling with how to address that question… I wouldn’t have done that before CPE. I would have taken a back seat. I was much more proactive than I would have been in the past."*

CPE helped them become more adept at communicating and asserting the chaplain’s contribution to whole-person care with greater clarity.

"*CPE has really taught me that you can be leadership within your own role… I think sometimes we’ve not always had the confidence to put our opinion out there … or what we can bring from the patient perspective. … bringing the spiritual aspect into it is about looking at the holistic approach. … And when we show that confidence, [the clinicians are] definitely keen to get us involved.”*

Furthermore, on the Chaplain Capabilities Scale, participants reported an improved ability to understand relational dynamics and collaborate effectively within chaplaincy, multidisciplinary, and other healthcare teams (Fig 4).

#### Reflective practice

**Fig 5:**
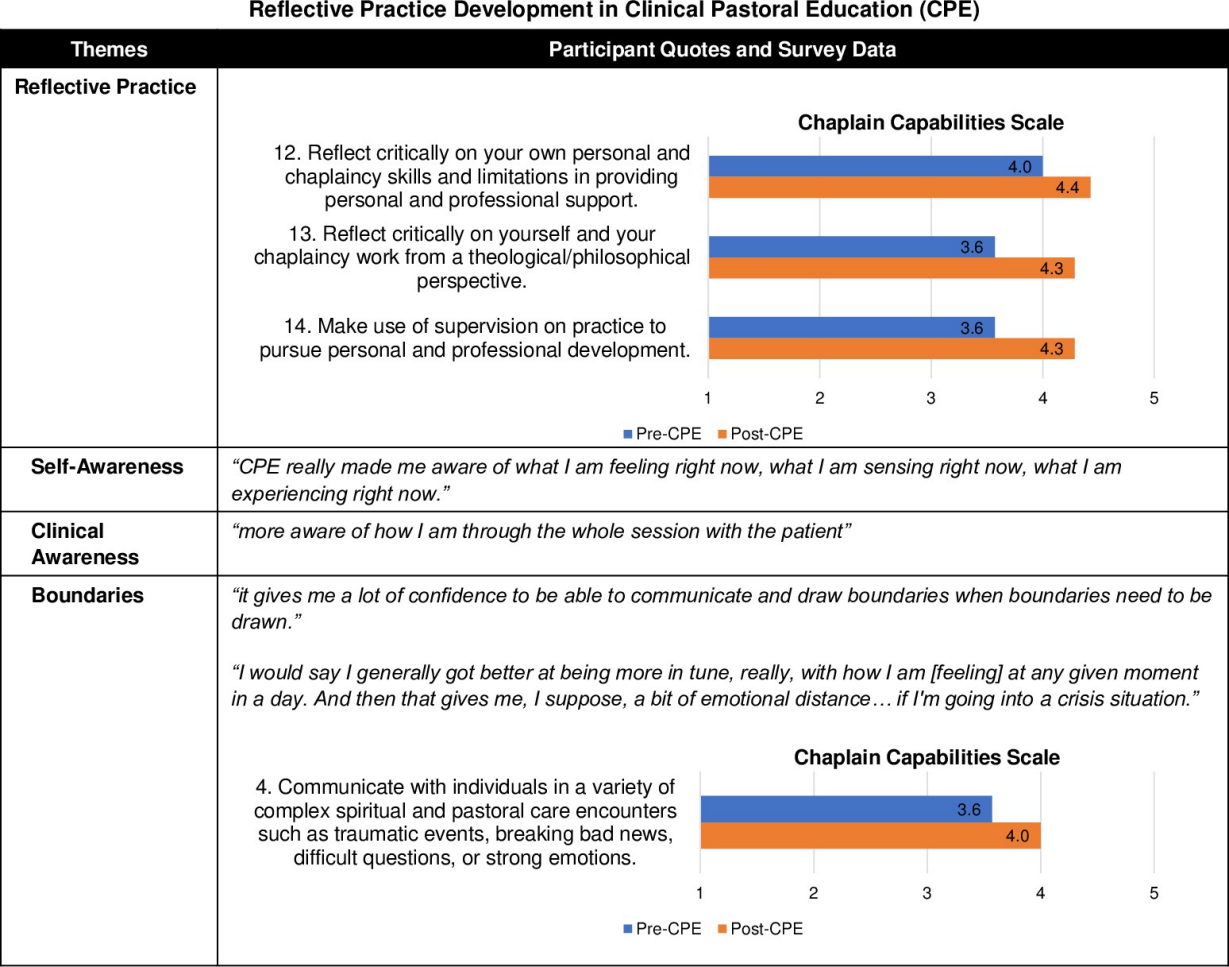
Reflective practice development in Clinical Pastoral Education (CPE). Fig 5 provides a joint display comparing qualitative themes with exemplary quotes and survey results with Chaplain Capabilities Scale (CCS) mean scores. The 14-item CCS was developed by the authors and was informed by the Spiritual and Religious Care Capabilities and Competences for Healthcare Chaplains by the UK Board of Healthcare Chaplaincy [31] and the ACPEI CPE Learning Outcomes [13].

CPE helped participants grow as reflective practitioners who integrate continuous reflection on themselves, their interactions with care recipients (i.e., patents, family, caregivers, staff, or others receiving chaplaincy care) and colleagues, and their spiritual care work. This development stemmed from the critical and in-depth reflection CPE asked students to do in their peer group and learning activities. Participants’ survey responses also showed an improved capacity to critically reflect on their own personal and chaplaincy skills and limitations, reflect on themselves and their chaplaincy work from a theological/philosophical perspective, and use supervision on practice to pursue personal and professional development (Fig 5).

*Self-Awareness*. CPE processes fostered participants’ personal and professional self-awareness. They were learning about the deeper layers of their personal histories, feelings, personhood, and what they brought into their spiritual care practice. Growing self-awareness had a widespread influence on participants’ interpersonal, clinical, and team functioning as chaplains. As a participant explained:

"*If I didn’t have this deep self-awareness that I have gained in CPE, I would be less attentive in a way with other people in the room. … CPE really made me aware of what I am feeling right now, what I am sensing right now, what I am experiencing right now.*"

As a key impact of CPE, a participant described their process exploring the complexities of their personal stories and how it related to care recipients.

"*There are characters and people in our stories. From people we see, we relate back to our stories, don’t we? I was made more aware of those connections and feelings that I get that are part of me. … I’m much more aware of how my story impacts how I relate to others in certain circumstances.*

Participants carried their growing self-awareness into their spiritual care practice, leading into their clinical awareness: "*[CPE] was challenging in terms of self-awareness and finding out those things about you that you do take into the room that you didn’t realise you took into the room when you see people*.*"*

*Clinical Awareness*. A common view among participants was that CPE increased their self-awareness to produce a clearer understanding of why they do what they do as chaplains and how those actions impact their care recipients. It helped them become *"more aware of how I am through the whole session with the patient*.*"* It improved their capacity to reflect on emotions (in themselves and others), spiritual concerns, and interpersonal dynamics more easily in clinical care, helping them to be more deliberate in their interventions. A participant described:

"*[I]t was about being very self-aware of how I am when I’m going to visit a patient, or how I’m with a colleague in terms of my attention span or what might be said across my face. Just making myself really aware of … what I am going into, and how I am responding."*

Another participant noted how clinical self-awareness enhanced reflective practice when they sought consultation on a particularly difficult spiritual care case.

"*… pre-CPE, [I] would have just talked about the clinical practice I did …. Whereas this time, we talked about the routine, the clinical practice, but also, I felt I knew the benefit of thinking about how this had impacted me emotionally and therefore how it shaped me. I just felt much more self-aware about how I felt with the [care recipients]. … Self-awareness is a really helpful thing for me to be more robust in tense situations as they arise."*

*Boundaries*. CPE participants noted their increased awareness and ability to create appropriate professional and personal boundaries.

"*[T]he cycle that I am experiencing is [that] I was able to identify and acknowledge of what I am bringing as a person into the chaplaincy, then it gives me a lot of confidence to be able to communicate and draw boundaries when boundaries need to be drawn."*

Another participant described that learning emotional self-differentiation enhanced their capacity to be collected and respond in crisis situations:

"*I would say I generally got better at being more in tune with how I am [feeling] at any given moment in a day. And then that gives me, I suppose, a bit of emotional distance. If I’m going into a crisis situation, then maybe I’m more … grounded in how I am, and therefore better able to respond, I think, probably, from a place of calmness within myself."*

Others also commented on their increased capacity to maintain boundaries in emotionally intense situations. This allowed them to be more effective in the moment and then in caring for themselves afterwards. Furthermore, survey results reflected respondents’ increased competence in communicating with individuals in a variety of complex spiritual care situations that involve traumatic events, breaking bad news, difficult questions, or strong emotions.

#### Emotional intelligence

**Fig 6:**
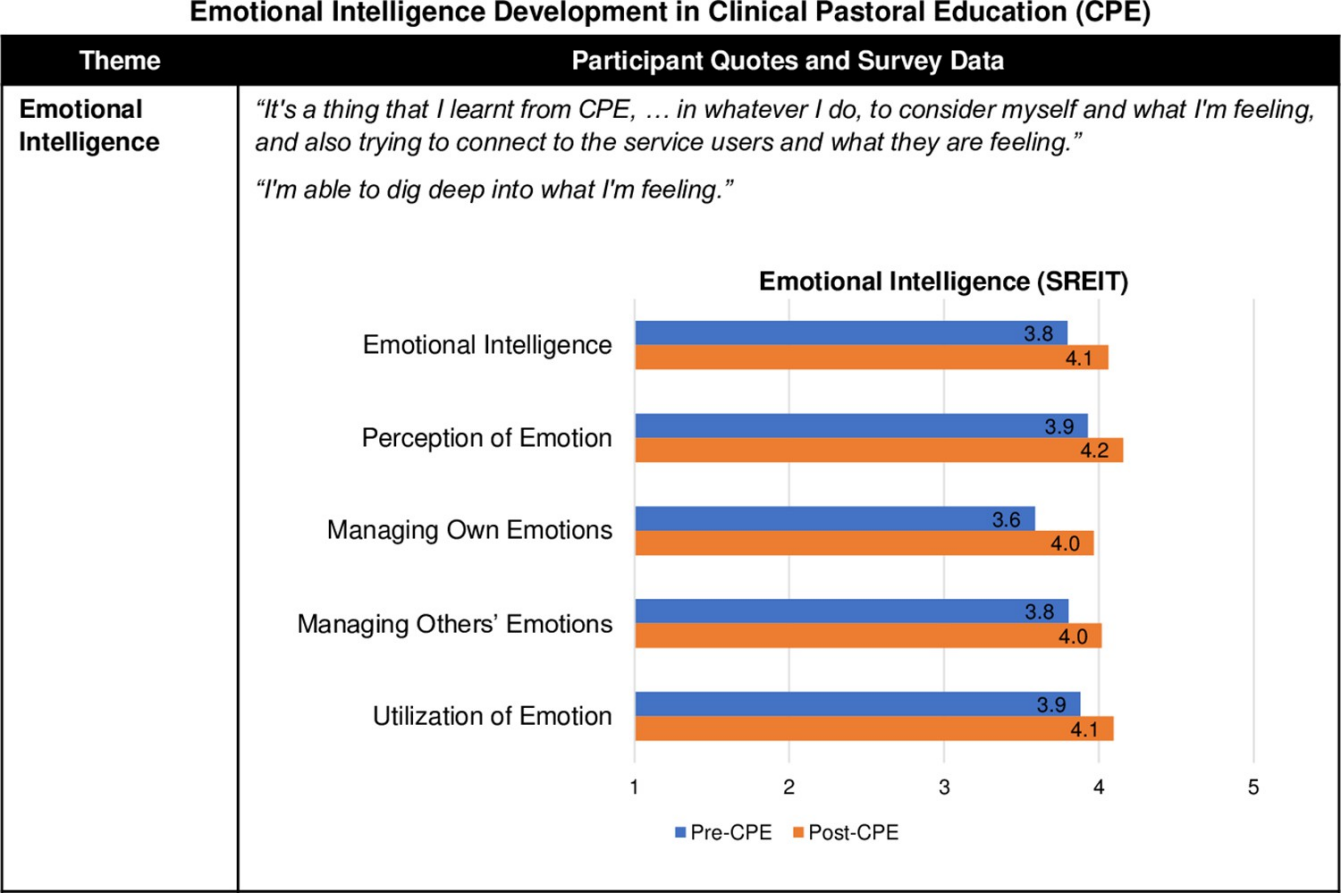
Emotional intelligence development in Clinical Pastoral Education (CPE). Fig 6 provides a joint display comparing qualitative themes with exemplary quotes and survey results with Self-Report Emotional Intelligence Test (SREIT) mean scores. The Self-Report Emotional Intelligence Test (SREIT) results, for overall emotional intelligence and four subscales, were calculated by using mean scores to allow comparison of scales with varying numbers of items [26, 27].

Participants described a substantial development in their emotional intelligence (EI)–the capacity to perceive, utilise, understand, manage emotions in self and others [35] (Fig 6). They commonly highlighted the application of their EI skills in their clinical work. *“It’s a thing that I learnt from CPE*, *… in whatever I do*, *to consider myself and what I’m feeling*, *and also trying to connect to the service users and what they are feeling*.*”* Furthermore, self-awareness, clinical awareness, and emotional intelligence were strongly interconnected in enhancing CPE participants’ chaplaincy practice. A participant reflected the impact of gaining the critically important skill of being in touch with their emotions, combined with their reflective practice: *"Self-awareness is the big thing … with the intense situations that I’ve been in*. *I’m able to reflect better afterwards … because I’m able to dig deep into what I’m feeling or what I did feel at the time*.*"*

Increased emotional intelligence was also evidenced by respondents’ improved SREIT scores, moving from 125.3 to 134 total scores (7%; 3.8 to 4.1 average scores. SREIT subscales showed that respondents had the greatest gain (11%) in the Managing Own Emotions domain, followed by 6% each on the Perception of Emotion, Managing Others’ Emotions, and Utilization of Emotion subscales (Fig 6 and S2 Table).

#### Listening and attending skills

CPE participants learnt to listen and attend to individuals’ feelings and stories more effectively and help them explore at a deeper level. Participants highlighted they made progress with their listening skills, especially their ability to create a safe space and *“not to be afraid of feelings*, *to listen to them and to stay*.*”* CPE fostered their capacity to remain present with the emotions, distress, and painful experiences of others more fully rather than trying to fix these emotions or move away from them.

"*[In CPE], we were being encouraged to listen to the emotion that was being expressed. … To stay with the emotion is what we became quite good at in our IPG sessions. Obviously, not rushing to fix the problem, but to stay with it. I found that I am really looking for that when I am with service users now. I’m thinking about the emotion and whatever that emotion is. I’m acknowledging that, and staying with it, and giving it space rather than moving on to something else. I think that is a big takeaway for me."*

Another participant described gaining more skills to deepen spiritual care conversations and help clients explore their thoughts and feelings by attending and using intentional silence:

"*I got bolder allowing more silence. I kind of stopped myself sometimes from asking even quite good open-ended questions. I realised if I waited longer, often giving them that time and space enabled them to find their way forward within the conversation rather than me thinking I had to help them, which might have taken them off in a direction you didn’t particularly want to go. … It is now much more a part of my practice to just wait a bit longer, rather than feeling like I’ve got to step in to help somebody.”*

**Fig 7:**
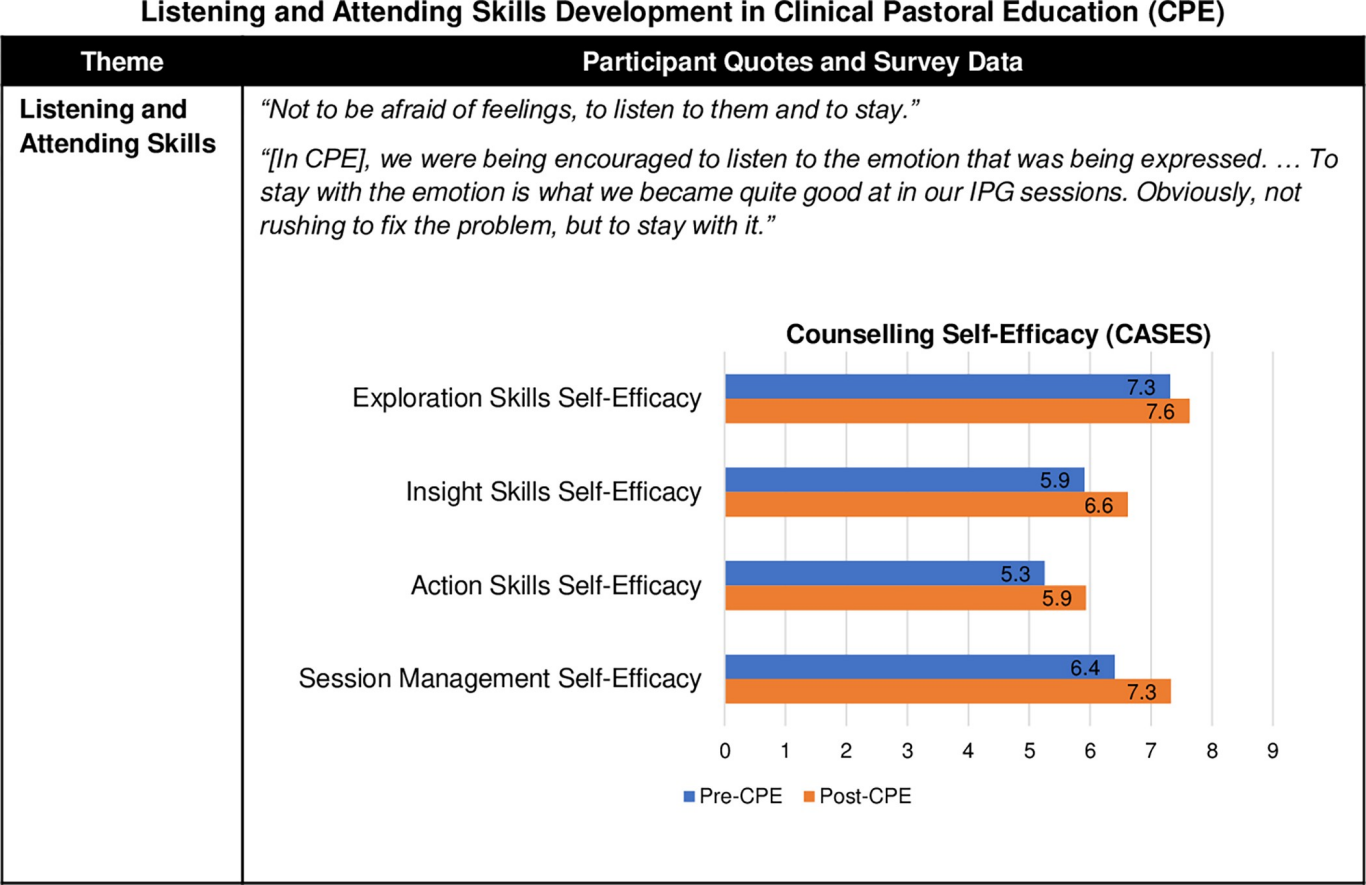
Listening and attending skills development in Clinical Pastoral Education (CPE). Fig 7 provides a joint display comparing qualitative themes with exemplary quotes and survey results with the mean scores of the Counselor Activity Self-Efficacy Scales (CASES) Part 1 and Part 2 [28].

Respondents’ counselling self-efficacy increased on all CASES scales (Fig 7), which confirmed their development in this area. They had the largest gains on the Session Management Self-Efficacy subscale (15%). This scale assessed skills to help clients explore their thoughts, feelings, and concerns and consider goals and actions. It also ascertained respondents’ capacities to know what to say next, respond with the best helping skill, keep sessions on track and focused, and remain aware of their intentions, purposes, and interventions. This was followed by gains in Action Skills Self-Efficacy to employ direct counselling interventions (13%) and Insight Skills Self-Efficacy to help clients better understand their thoughts and feelings (12%). The smallest increase (4%) was in the Exploration Skills Self-Efficacy to ask open questions, listen, reflect feelings, restate, use intentional silence, and attend, which already had the highest baseline (7.3) and still reached the highest post-training levels (7.6).

#### Diversity in chaplaincy care

**Fig 8:**
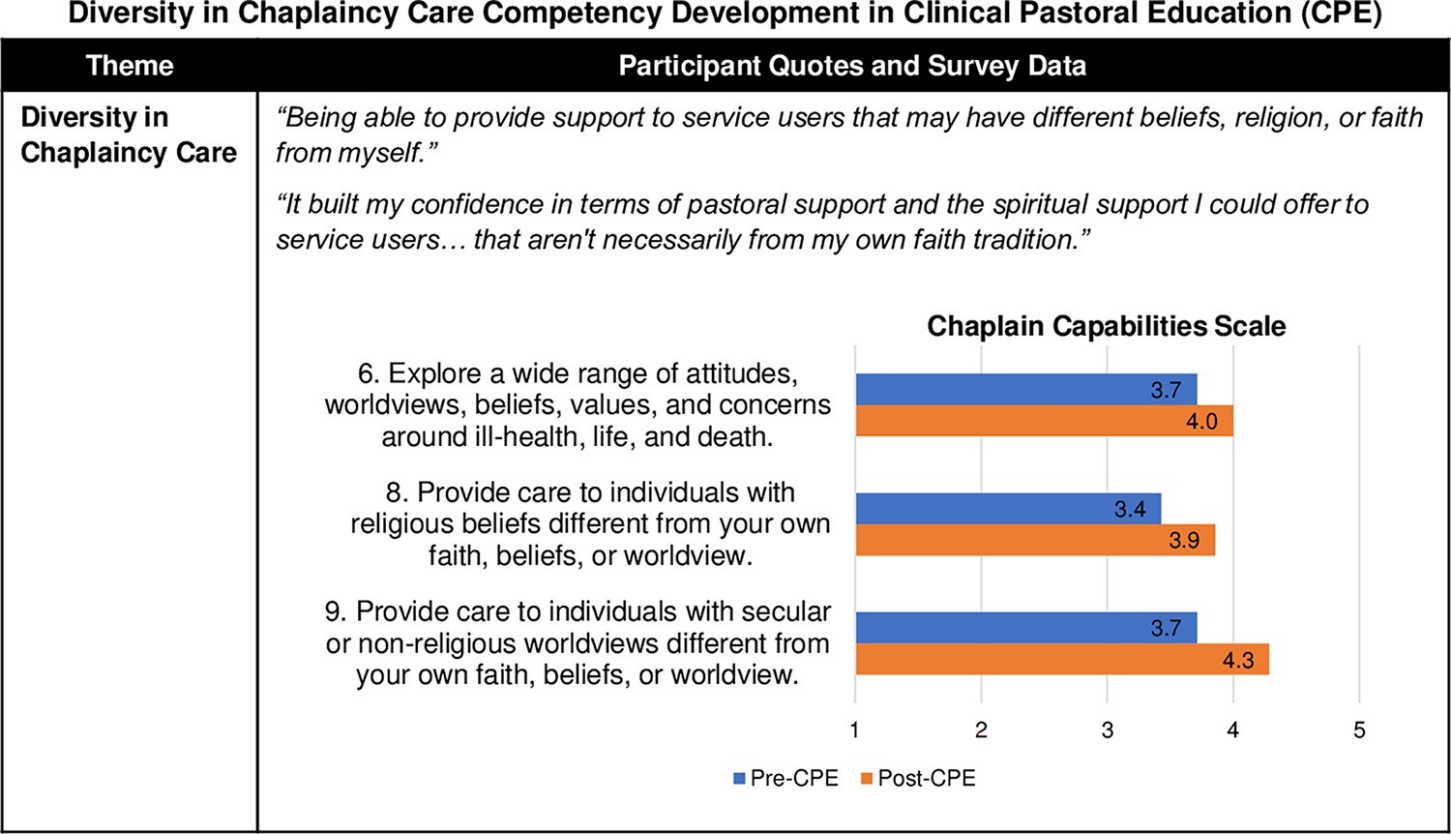
Diversity in chaplaincy care competency development in Clinical Pastoral Education (CPE). Fig 4 provides a joint display comparing qualitative themes with exemplary quotes and survey results with Chaplain Capabilities Scale (CCS) mean scores. The 14-item CCS was developed by the authors and was informed by the Spiritual and Religious Care Capabilities and Competences for Healthcare Chaplains by the UK Board of Healthcare Chaplaincy [31] and the ACPEI CPE Learning Outcomes [13].

Participants agreed that CPE greatly increased their ability *“to provide support to service users that may have different beliefs*, *religion*, *or faith from myself*.*"* Consistent with the survey responses (Fig 8), this theme entailed their growing capacity to explore a wide range of attitudes, worldviews, beliefs, values, and concerns related to health and to provide effective spiritual support to persons with diverse faith or secular/non-religious perspectives and different beliefs or philosophies from their own. This area also included improving collaboration with appropriate chaplains or faith leaders concordant with the individual’s faith or worldview when needed. Participants with different religious and non-religious identities grew in this area:

"*I think it is connecting on a human level with everybody, because I’m non-religious. I did struggle a little bit with people of faith. If I take that away, I can connect as a human being and add that in after as it were. I found that I’m much better at that now than I was."*

"*[B]efore CPE*, *if there were any Muslim patients around*, *without ministering to them and listening to them*, *I would have told them that we do have Muslim chaplains and would they like me to refer them*. *Now*, *I would listen to them and*, *if necessary*, *refer*. *It was a huge shift for me*.*"*

Participants also identified the diversity of the CPE group as a catalyst for gaining confidence in providing spiritual care to persons with diverse religious and non-religious backgrounds.

"*There were lots of conversations about Muslim patients from people on the team asking questions, probably because I was a Muslim in the [CPE] group. For me, the flip side of that is how much I was learning from my colleagues. … I think, what made it so unique was that we were a mixed group. … It built my confidence in terms of pastoral support and the spiritual support I could offer to service users… that aren’t necessarily from my own faith tradition. It gave me that little bit more confidence actually, that it’s okay for me to do that."*

#### Spiritual assessment

**Fig 9:**
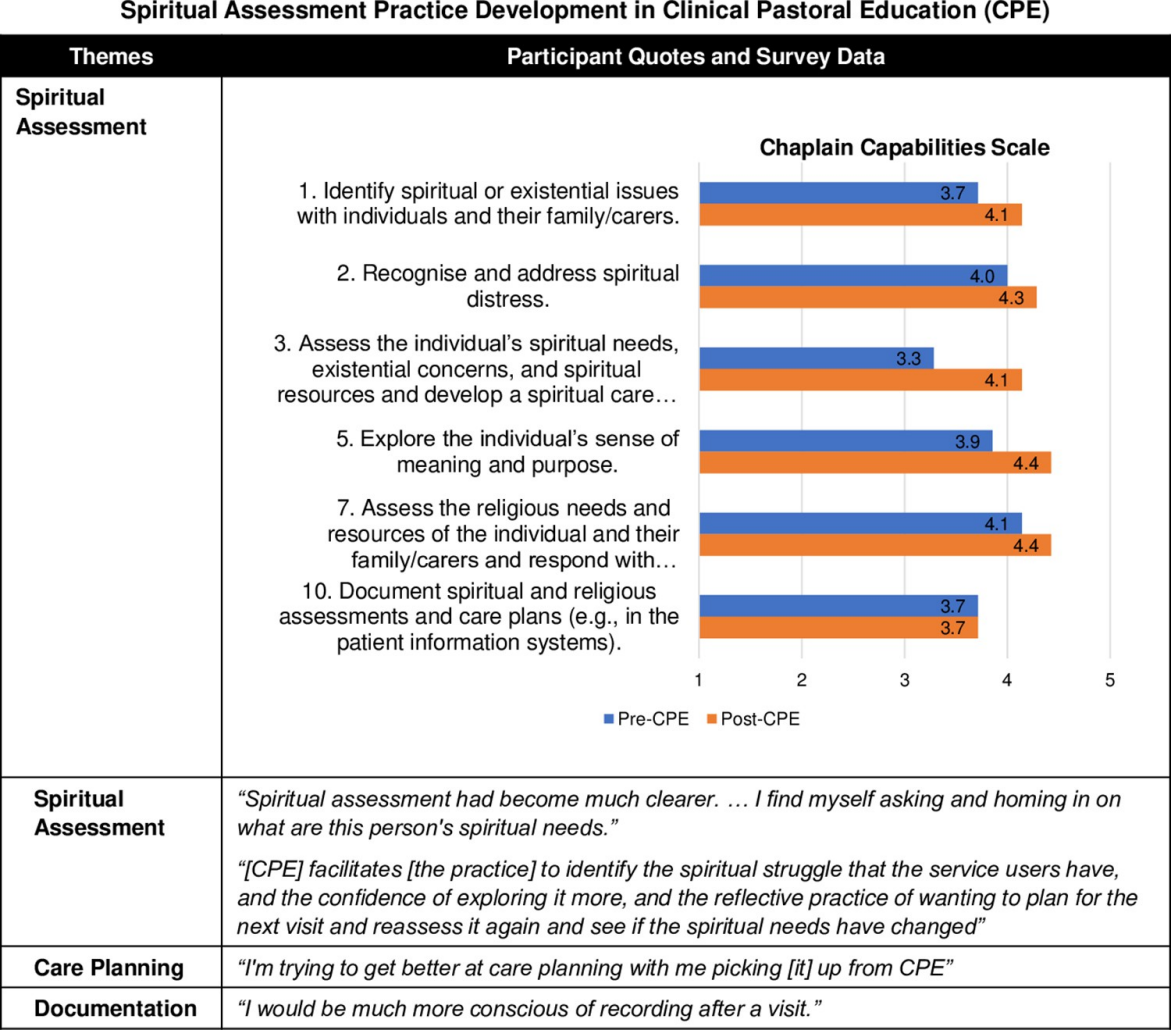
Spiritual assessment practice development in Clinical Pastoral Education (CPE). Fig 9 provides a joint display comparing qualitative themes with exemplary quotes and survey results with Chaplain Capabilities Scale (CCS) mean scores. The 14-item CCS was developed by the authors and was informed by the Spiritual and Religious Care Capabilities and Competences for Healthcare Chaplains by the UK Board of Healthcare Chaplaincy [31] and the ACPEI CPE Learning Outcomes [13].

CPE improved participants’ competencies in conducting spiritual assessments, formulating ongoing care plans, and documenting spiritual care. They developed more robust skills and language to identify and address spiritual struggle. Survey results further showed gains in several skills related to spiritual assessment (Fig 9), such as assessing spiritual or existential issues, resources, and spiritual distress, exploring individuals’ sense of meaning and purpose, and developing spiritual care plans. Interestingly, another item on the ability to document spiritual and religious assessments and care plans did not show pre-post change, in contrast with qualitative data.

A participant explained that discussing clinical experiences and cases in the CPE group *“facilitates [the practice] to identify the spiritual struggle that the service users have*, *and the confidence of exploring it more*, *and the reflective practice of wanting to plan for the next visit and reassess it again and see if the spiritual needs have changed*.*"* Another one echoed,

"*Spiritual assessment had become much clearer, and the question I’m asking myself and honing in on what are this person’s spiritual needs, can I identify them. In that sense, yes, [CPE has] altered my practice, in that I’m much more aware of actually asking myself that question during encounters. It’s always there."*

Furthermore, participants recognised the benefits of care planning and incorporated it into their practice:

"*Just trying [care planning] rather than going ad hoc and then seeing where it is going to go after the initial assessment. I’m trying to get better at care planning with me picking [it] up from CPE … for the sessions to be more productive."*

Moreover, some noted improvements in their practices of documenting their care, *"I would be much more conscious of recording after a visit*.*"*

### 2. Catalysts for development in CPE

**Fig 10:**
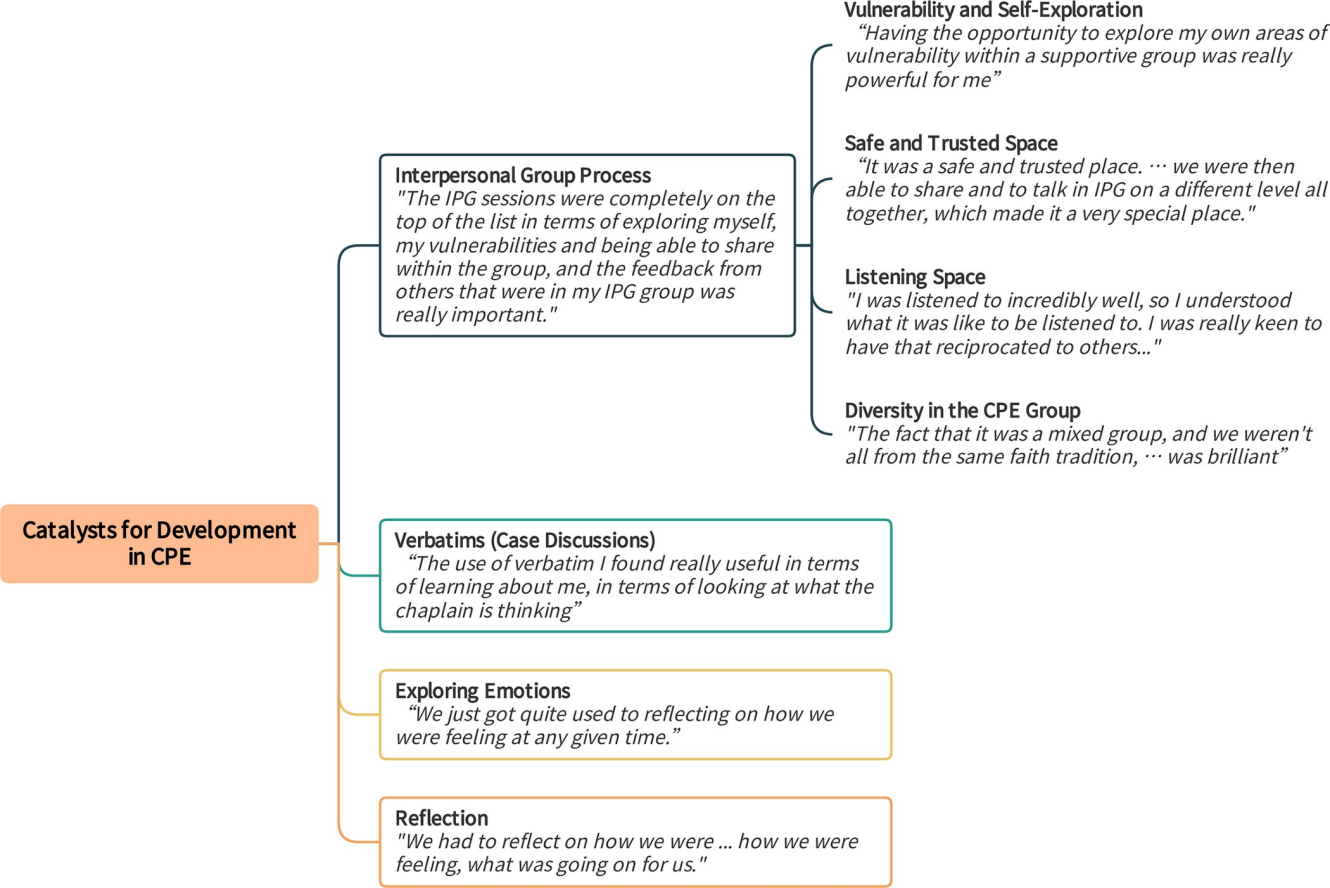
Catalysts for development in Clinical Pastoral Education (CPE). Fig 10 provides themes and subthemes with exemplary quotes regarding Catalysts for Development in Clinical Pastoral Education (CPE).

In this overarching theme, participants highlighted several factors as catalysts for change, learning, and development in CPE. In Fig 10, themes represent elements of the CPE process and instruction which fostered growth in a range of areas, including but not limited to the developmental themes presented above. For example, encouraging students to explore their vulnerabilities and feelings in the CPE group increased their self-awareness and emotional intelligence personally and in interactions with others as chaplains. Additionally, engaging diverse viewpoints and beliefs of CPE peers in the learning group directly improved participants’ chaplaincy skills to serve people with different beliefs than their own.

#### 2.1. Interpersonal group process (IPG) in CPE

Participants agreed with each other that the interpersonal group process of CPE (i.e., IPG) was instrumental in their development. One participant said, "*the IPG sessions were completely on the top of the list in terms of exploring myself*, *my vulnerabilities*, *and being able to share within the group*. *And the feedback from others that were in my IPG group was really important*." The group provided the relational context with peers and the supervisor, in which they observed and practised working with vulnerabilities, feelings, interpersonal dynamics, and listening. They experienced being listened to and received feedback and consultation. They applied their learnings from the group setting to their professional identities and interactions as chaplains. "*Within the group*, *you are able to have a sense of awareness and part of your identity as a chaplain*, *but it has to be done in a group*.*"*

*Vulnerability and Self-Exploration*. The group sessions encouraged participants–and even challenged them–to explore areas of their vulnerabilities, identities/self, and life stories: *"having the opportunity to explore my own areas of vulnerability within a supportive group was really powerful for me*.*"* It also taught them to skilfully help patients do the same in the clinical context.

"*Things that I have put away for a very long time, that I have not wanted to deal with and stored in these boxes, which are really dusty. [I] was to be able to bring them to the forefront of my vulnerability … and think about what that means for me and how I’m dealing with that. [It] equip[ed] me to be able to take 100% of me to whomever else I am seeing as well. It was a tough one, but I think it was the best part of the process for me. It helped become a healing journey for myself."*

Connecting with oneself more deeply allowed participants to connect with and be available to care recipients more fully:

"*CPE … allowing me and giving a space for me to connect to my deeper self and bring that into the ministry that I do or the chaplaincy work that I do. That is very helpful because then I become a gift to them in my limitations, and for them as well, to have this full attention that they deserve to have at these moments of their life. Because I am in touch with what I am feeling at that moment."*

*Safe and Trusted Space*. Participants felt that the expectation of vulnerability was challenging at first, but having a safe and trusting group environment allowed them to lean into the group process.

“*It was challenging in terms of the first week we didn’t know one another, did we, at all, in any way shape or form. Me personally, I didn’t really know what to expect either at all. The fact that you share your story from week one was challenging from the offset really. … It was a safe and trusted place to do that, and it stayed a safe place. … From that first week, when we really shared our stories, we were then able to share and to talk in IPG on a different level all together, which made it a very special place. Challenging but safe.”*

*Listening Space*. The development and practice of listening skills in IPG was crucial for the students. They learnt to listen to their peers and learnt from being listened to and observing how group members listened to each other. They directly carried over these developing skills to their clinical practice.

"*IPG sessions, which is where we had a chance to talk and listen to each other. I think that was a really helpful environment for us to try out our listening skills to each other. I think they improved weekly. … I personally felt, anyway, I was listened to incredibly well, so I understood what it was like to be listened to. I was really keen to have that reciprocated to others to try and hold our space, and hold some silence, and stay with somebody’s feelings in the group, so that we could help them to feel heard. That [has] definitely come over into my clinical practice. I think my clinical skills [and] my listening skills have improved.”*

*Diversity in the CPE Group*. Participants understood that engaging the diversity of CPE group members helped them develop their capability and confidence to support care recipients with diverse beliefs and backgrounds.

"*I also think the fact that it was a mixed group, and we weren’t all from the same faith tradition, … was brilliant in terms of how it was structured. … It actually confirmed a lot of things that we might not even thought of in the past. We don’t need to be chaplains to our own so to speak. I think it just gives a little bit more confidence in terms of some of the generic pastoral work that we do as well."*

Hearing each other reflect on their different beliefs, theologies, or philosophies cultivated a greater understanding of diverse viewpoints. "*It also really helped to actually look at my peers’ theological reflections as well and helped greatly in understanding other sources of wisdom and other traditions as well*.*"*

#### 2.2. Verbatims (case discussions)

Discussing clinical cases and receiving feedback from the supervisor and peers (i.e., verbatim sessions) helped participants learn about themselves as chaplains and critically evaluate and expand their clinical skills. *"The use of verbatim I found really useful in terms of learning about me*, *in terms of looking at what the chaplain is thinking*, *what the chaplain is doing*.*"* Another participant added:

"*Hearing from the peer in the verbatim the different approach of providing support is very helpful to equip me with skills that are useful every time I go and visit the patients. And because of that, it gives me more confidence, and validation, as well."*

#### 2.3. Exploring emotions to build emotional intelligence (EI)

Participants were encouraged to explore and work with emotions in themselves and others during the IPG and verbatim sessions. This approach fostered participants’ EI, which they put into practice in spiritual care. They made a clear connection between gaining EI in the CPE group and increasing EI in chaplaincy practice.

"*Throughout our sessions, was it the beginning and end of each day [our CPE supervisor] would ask us how we are feeling? And so, we just got quite used to reflecting on how we were feeling at any given time. And he would push us sometimes, too. If we came out with a fairly banal word, he would say, ’So what does that mean?’ and would push us a bit. So, I think I would say I generally got better at being more in tune, with how I am at any given moment in a day."*

#### 2.4. Reflection

CPE spurred participants to reflect and enhance their reflective capacities, preparing them as reflective practitioners: *"We had to reflect on how we were*… *how we were feeling*, *what was going on for us*.*"* As a source of growth, participants highlighted the vigorous critical reflection on their identities, belief systems, life stories, relationships, and spiritual care interactions that occurred in group sessions and learning activities.

"*I think it helped me to probably be more aware than I was before CPE of how I am when I go into an encounter because, when we were doing the verbatims, we had to reflect on how we were, where, what time of the day it was, how we were feeling, what was going on for us."*

### 3. Advantages of CPE for chaplaincy education

**Fig 11:**
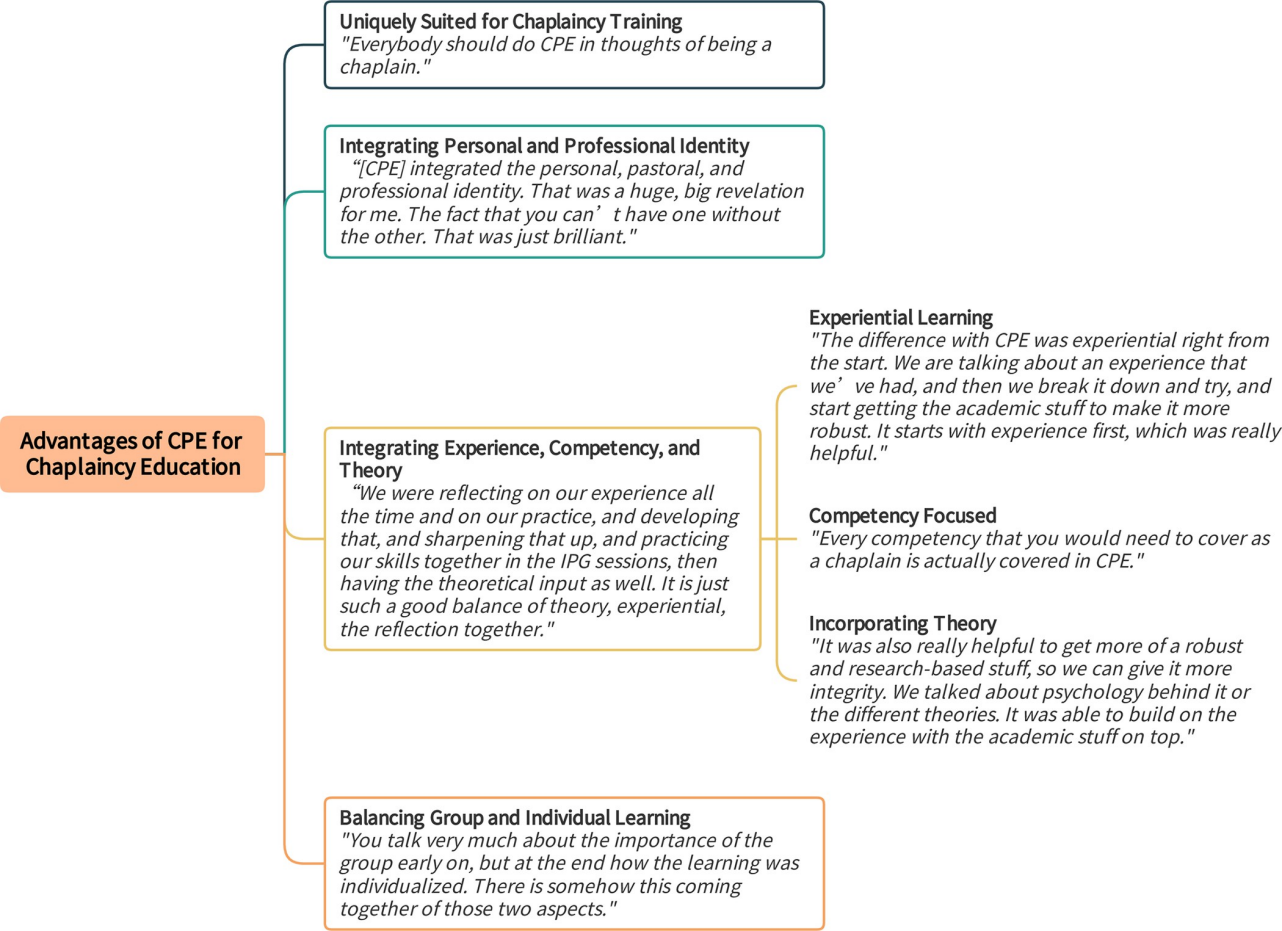
Advantages of Clinical Pastoral Education (CPE) for chaplaincy education. Fig 11 provides themes and subthemes with exemplary quotes regarding Advantages of Clinical Pastoral Education (CPE) for Chaplaincy Education.

Participants highlighted characteristics that made CPE a distinctively robust and effective method for chaplaincy education (Fig 11), compared to other types of chaplaincy training they had experienced.

#### 3.1. Endorsing CPE as uniquely suited for chaplaincy training

Participants unanimously endorsed the sentiment that "*everybody should do CPE in thoughts of being a chaplain*." They added that CPE may equally benefit novice and experienced chaplains: “*it’s a really valuable tool for people coming into chaplaincy*. *… From my perspective*, *I also think it’s invaluable for more experienced chaplains*.” Notably, some of this cohort were experienced chaplains and some were relatively new to chaplaincy, yet all felt they had developed as chaplains and benefited in their clinical practice because of CPE.

Moreover, participants were eager to identify aspects that made the CPE model different and uniquely beneficial.

“*CPE brought something completely different to chaplaincy and exploring. … Because it really brings insight into how we manoeuvre with the patients and how we get down to a deeper level, rather than just going in, asking if they would like prayers or not like prayers, or about rulings. … It can completely change the focus on getting people to think about what chaplaincy is about.”*

Additionally, results on the post-CPE Net Promoter Score (NPS) item underscored this sentiment. All responses fell in the “promoter” category when asked how likely it is that they would recommend CPE to their chaplain colleagues or others in chaplaincy training (six rated 10 and one 9). It produced an NPS of 100, a valid indication of not only a high level of satisfaction but also a strong enthusiasm for the value of CPE for others.

#### 3.2. Integrating personal and professional identity

Participants concurred that CPE simultaneously addressed the interdependent domains of personal identity and who they were as professional chaplains: "*the individual*, *the pastoral*, *the professional identity was really well balanced in a way that I don’t think exists in other training courses*.” They recognised this element as instrumental for chaplain development. Participants saw this interplay as a distinctive benefit of CPE over other educational models.

"*[CPE] integrated the personal, pastoral, and professional identity. That was a huge revelation for me, the fact that you can’t have one without the other. That was just brilliant. The fact that I had to bring my personal identity into this, because I was able to become more self-aware and think about my personal identity, I was able to carry that across as a pastoral practitioner. It also made me more confident in my professional identity."*

#### 3.3. Integrating experience, competency, and theory

Participants viewed CPE as a comprehensive training model because it leveraged experiential learning, built competencies, and incorporated relevant theories for chaplaincy. These aspects worked together to promote participants’ growth.

"*I really valued the fact that we were reflecting on our experience all the time and on our practice, and developing that, and sharpening that up, and practicing our skills together in the IPG sessions, then having the theoretical input as well. It is just such a good balance of theory, experiential, the reflection together."*

Fig 11 provides quotes to illustrate each of the experiential learning, competency building, incorporating theory aspects of CPE.

#### 3.4. Balancing group and individual learning

Participants pointed to CPE’s advantage of utilising both small-group learning and individualised learning. *"You talk very much about the importance of the group early on*, *but at the end how the learning was individualised*. *There is somehow this coming together of those two aspects*. *It seems to be key in the learning*.*"* Another participant added:

"*You can’t have one without the other. You are able to develop your own individual identity because of the group, but then also the dynamics of the group really improve, if we share our own identity as well. They both go together. That is what works really well."*

### 4. Participant experiences with CPE course structure

**Fig 12:**
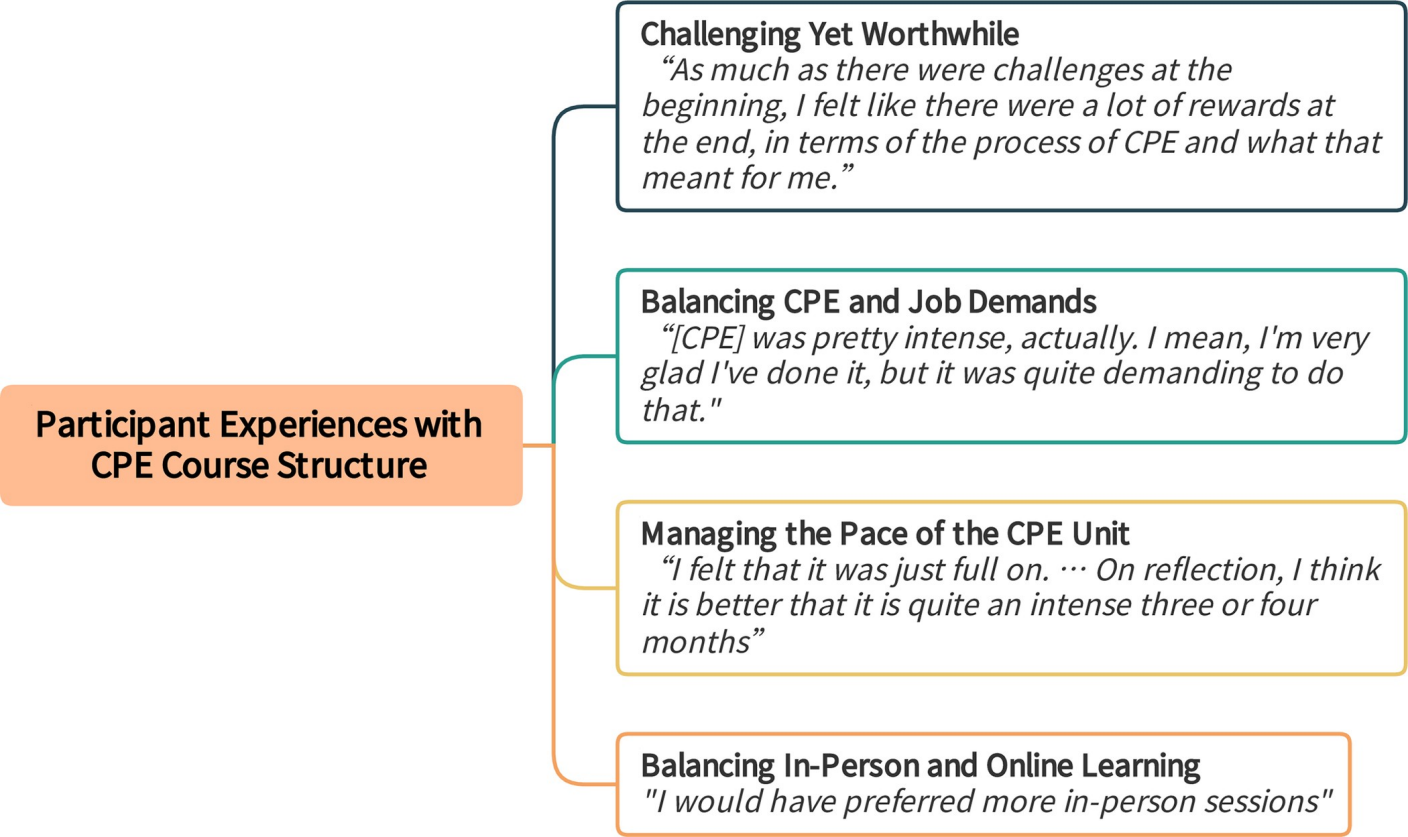
Participant experiences with Clinical Pastoral Education (CPE) course structure. Fig 12 provides themes and subthemes with exemplary quotes regarding Participant Experiences with Clinical Pastoral Education (CPE) Course Structure.

Participants were highly satisfied with their CPE experience. However, they expressed a range of opinions regarding the CPE unit’s structure and delivery as they all were undertaking CPE alongside their existing professional roles (Fig 12).

#### 4.1. Challenging yet worthwhile

Participants said that the CPE course felt both challenging and worthwhile. They perceived the challenges more keenly as they began the process. However, they gradually understood how to make use of those elements. For instance, the IPG group process was seen as especially challenging and new at the beginning but ultimately became a powerful vehicle for transformational learning: "*challenging but safe*,*"* as a participant put it.

"*As much as there were challenges at the beginning, I felt like there were a lot of rewards at the end, in terms of the process of CPE and what that meant for me. At the beginning, I thought, ’I don’t think I’m going to last very long in this process.’ Interestingly, I came out completely different at the other end. I think it really helped. It came with its challenges but with its rewards too."*

#### 4.2. Balancing CPE and job demands

Participants underscored that CPE required high-level commitment and workload, which was challenging to fit with the competing demands of their full- and part-time jobs during CPE.

"*[I]t was just juggling and keeping up with the demands with my job at the same time as the demands of the course. It was pretty intense, actually. I mean, I’m very glad I’ve done it, but it was quite demanding to do that."*

Some participants also shared the sentiment of "*I had no idea*," meaning that, not having done CPE before, they could not have fully and accurately anticipated the extent of work and energy involved in the CPE unit. The participant continued, *"it probably would have been better for me if I would have just had a clearer idea in my mind of the degree of commitment that was needed*, *because it was a high degree of commitment really*.*"*

#### 4.3. Managing the pace of the CPE unit

Participants shared their views on the advantages and disadvantages of the CPE unit’s pace. They felt that, while extending the unit over a longer period could have eased the pressure, they were not convinced it would have been beneficial.

"*I felt that it was just full on. It was relentless one week, and then we would have to do another written work as soon as we handed in one more. I think we suggested that it would be great if it was twice as long…. There are pros and cons to that, because then it would just be drawn out for far too long. I think maybe you would get really fed up with being in CPE for such a long time. On reflection, I think it is better that it is quite an intense three or four months."*

In fact, some felt that the fast pace and structure kept them engaged and motivated and prevented the course from dragging on unnecessarily.

" *I’m glad we did it the way that we did it. I can’t believe I am saying that. I just feel like that structure makes such a big difference. … it was challenging, but if it was stretched out any longer, we would have still had the same amount of work to do. … And I don’t think the impact would have been the same. There were challenges that came with it. It worked."*

#### 4.4. Balancing in-person and online learning

Although acknowledging online sessions enabled them to participate from different locations, participants found long online sessions to be taxing. Moreover, they agreed with the opinion: *"I would have preferred more in-person sessions*,*"* whereas noted travelling can be challenging. Participants thought that meeting in person was especially beneficial at the beginning when relationships formed in the CPE group.

"*It’s really important that it has to be face to face around the first couple of days and face to face at the end of the whole class. It is really important that we are all together when we are sharing our personal stories right at the start. I think it would have been really difficult online."*

Ultimately, participants agreed that there is not a perfect solution for the course’s pace and meeting patterns. “*It’s just hard to fit in that number of hours of training over however many weeks it is*, *so I don’t think there’s any easy answer*.”

## Discussion

This study investigated the impact of participating in a CPE unit on chaplains’ development. Furthermore, we explored participants’ views on their learning experiences, with a particular focus on development, educational factors, the CPE model, and course structure. Quantitative and qualitative findings converged to provide rich evidence that CPE generated personal and professional development among chaplain participants.

### Development pathways

Data revealed participants’ perceptions of their development along various pathways: confidence, reflective practice, emotional intelligence, listening and attending skills, diversity in chaplaincy care, and spiritual assessment. Participant responses on pre-post surveys confirmed growth in these areas by showing gains in chaplain capabilities, emotional intelligence, and counselling self-efficacy from before to after CPE.

These results are consistent with several studies. Vanderstelt et al. [21] found most students reported a lasting positive impact of CPE even years after their CPE experiences in Canada. They identified themes of growth such as self-awareness, emotional intelligence, personal growth with increased confidence, professional identity, self-care/boundaries, theological reflection, multifaith awareness, communication, listening, and assessment skills. Previous studies in the USA and Canada showed a similar interplay of personal and professional development in CPE [16, 20] in areas such as self-awareness [20, 22, 36], self-reflection [19], boundaries [20], emotional intelligence [19, 21], pastoral and clinical skills [17, 19].

Furthermore, emotional intelligence increase in our sample of CPE participants was more than double than the results of Jankowski et al. [19] (7% vs. 2.2%; measured by SREIT). Our participants’ gains in counselling self-efficacy were comparable to trainees in psychology [37], counselling [28], and social work [38] who participated in helping and counselling skills training (measured by CASES). However, these comparisons are limited since we did not test the statistical significance of changes because of our small sample size.

### Confidence, professionalism, leadership, and teamwork

CPE helped participants increase their self-confidence and translate it into confident professionalism, leadership, and teamwork in their chaplain roles. This development enhanced participants’ collaboration with multidisciplinary teams and chaplain colleagues. Moreover, it was evident that CPE promoted greater clarity about their chaplain role and contributions, leading to greater capacity to assert their value.

Our findings parallel the doctoral research by den Toom [39] examining changes among chaplains participating in the Dutch Case Study Project. This project involved a rigorous process of reflection and analysis of spiritual care cases and clinical practice combined with discussion of each other’s cases. Chaplains reported growth in their awareness and professional identity, made their knowledge more explicit, practised more reflectively, and functioned in a more purposeful, goal-oriented manner. Moreover, chaplains clarified and strengthened their professional identity and increased their self-confidence, agency, joy, and motivation in their practice [39, 40]. Further emphasizing the importance of these factors, an international expert panel on chaplain leadership during the COVID-19 pandemic found that chaplains’ ability to lead with professional confidence and build trusting relationships with healthcare colleagues and executives greatly determined the extent to which chaplain contributions were valued and integrated in their healthcare institutions [41].

### How chaplain development occurs in CPE

**Fig 13:**
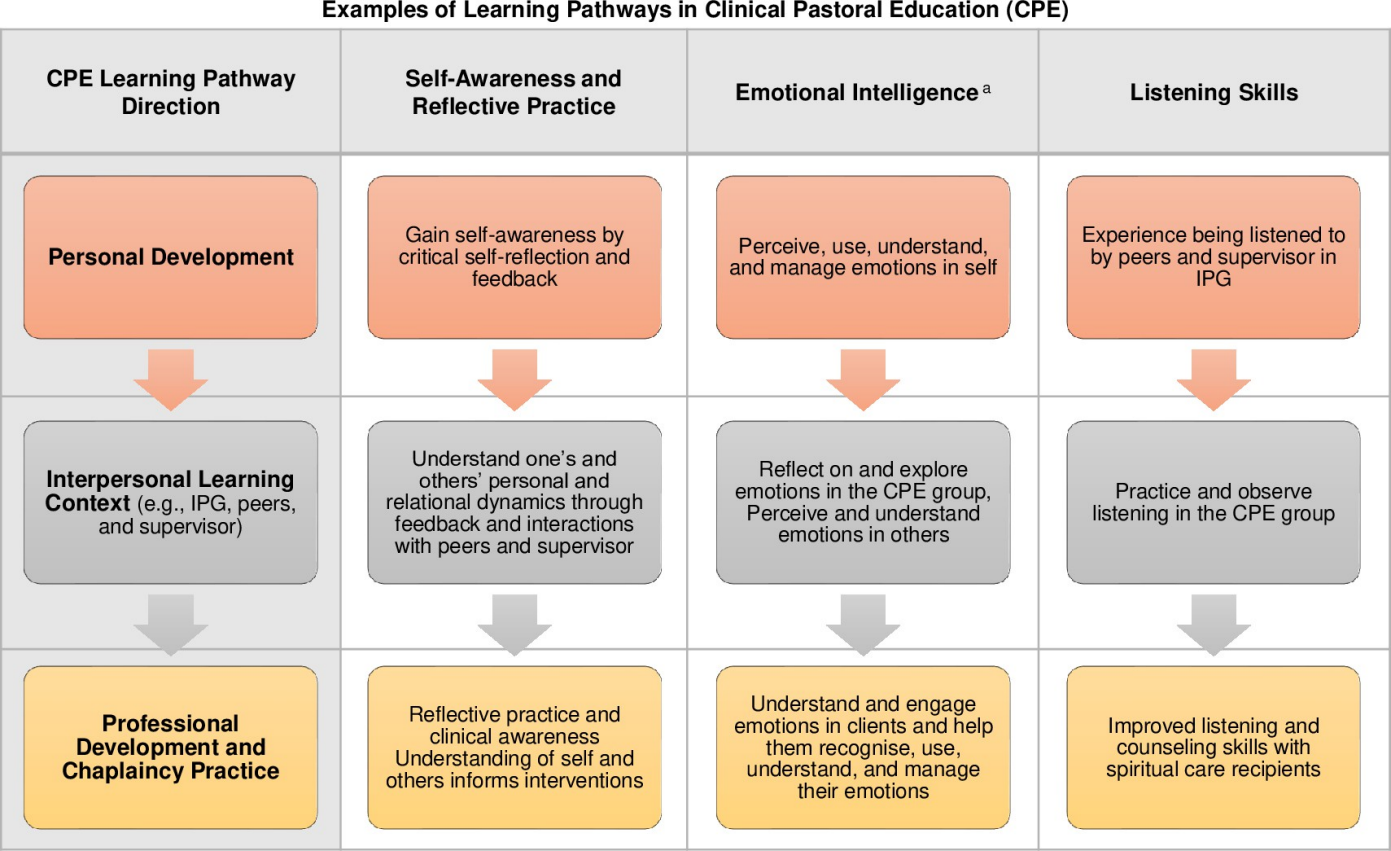
Examples of learning pathways in Clinical Pastoral Education (CPE). Fig 13 shows the conceptualisation of the learning pathways in CPE that moved from personal development, through the interpersonal learning context of CPE, and translated into chaplain competency and practice. ^a^ Emotional intelligence is understood based on the four-branch model of emotional intelligence of Mayer & Salovey [35].

Personal growth and professional development appeared to be interconnected and mutually reinforcing in most areas and directed toward enhancing chaplaincy practice. Findings suggest that general learning pathways moved from personal development, through the interpersonal learning context of CPE (e.g., IPG), and translated into chaplain competency and practice. To illustrate this underlying pattern, the learning processes could be conceptualised as shown in Fig 13.

For example, CPE enabled participants to expand their self-awareness. The aim was not only self-knowledge but also application of self-awareness, a keen awareness of the personal, relational, and spiritual/religious dynamics of others, and appropriate boundaries in professional helping relationships. Their growing self-awareness also enhanced participants’ capacity to continuously reflect on themselves, their practice, and their impact on others (i.e., reflective practice). Developing listening skills and emotional intelligence appeared to follow a similar process moving through the personal, interpersonal, and professional chaplaincy learning domains (Fig 13). The thematic map by Vanderstelt et al. [21] similarly underscored the interconnections between personal, relational, and spiritual care and therapeutic skills growth areas in CPE.

### Factors influencing chaplain development in CPE

We identified relevant determinants in three categories: catalysts for development, distinct advantages of the CPE model, and salient experiences with course structure, which collaborated to promote chaplains’ personal, professional, and competency development in CPE, as depicted in Fig 2. Participants highlighted growth-promoting factors that propelled learning in CPE: consistent practices of reflection, listening and feedback among group members, and examining emotions in the interpersonal learning context. The interpersonal group process, with the vulnerability, safety, and diversity experienced in that space, helped them develop self-awareness, emotional intelligence, listening and spiritual care skills, personal-professional integration, and reflective practice. Verbatims allowed participants to learn by receiving feedback from the supervisor and peers and by listening to their peers present cases, reflect, and receive feedback. These case discussions were essential to improve participants’ skills to assess and respond to spiritual concerns and become reflective practitioners.

Similarly, previous studies showed that CPE students underscored the effectiveness of experiential learning through clinical experience, interpersonal group process and supervision, and the use of verbatims [18, 20–22]. The combination of these elements appeared to be at the heart of the effectiveness and transformative nature of CPE.

### CPE for chaplaincy education in England

Participants’ experiences and views endorsed CPE as a robust and effective training model for chaplaincy. They unanimously recommended CPE for chaplaincy education across a range of experience levels, from those entering the profession to experienced chaplains, in the English context. The themes that highlighted the distinctive value and advantages of the CPE model for chaplaincy education further substantiated their recommendation. These themes included integrating personal and professional identity, integrating experience, competence, and theory, and balancing group and individual learning, all within a multi-belief and multicultural context. It was apparent that participants recognised the characteristics that made CPE worthwhile and transformative for them and would make it so for most chaplains. Similarly, 94% of the participants recommended CPE to others in the Vanderstelt et al. (2023) study.

Finally, participants reported positive student experiences while acknowledging the challenging aspects of the CPE course structure: balancing the demands of CPE and their work responsibilities, the course’s pace, and in-person and online interactions. Parallel to participants’ opinions on online and in-person CPE learning, CPE educators and students the USA reported certain benefits of online or hybrid modalities: ensuring access to CPE; not reducing the quality of learning; and being as effective as in-person CPE [42–44]. However, they desired more in-person interaction and found online learning not without limitations [42, 43].

Questions for CPE research and implementation in the English healthcare context remain. Further research is required to investigate CPE’s effects and longer-term impact in a larger sample using mixed methods. Future studies will be essential to determine the benefits and effectiveness of CPE compared to other chaplaincy training models and control groups. However, it will become feasible only after more participants complete CPE units in England and are available to recruit in such studies. The present study added to the knowledge base and presented relevant pilot data to consider for the next phases of implementation and research in this area.

## Strengths and limitations

Our study employed an integrated mixed methods approach in a small yet diverse sample, with 100% of CPE students participating. Neither individual method was compromised by applying mixed methods; instead, they enhanced each other. The qualitative inquiry produced nuanced descriptions of learning in CPE. Quantitative measures helped establish clear relationships with validated constructs of EI and counselling self-efficacy and specific chaplain competencies (CCS), an approach that has historically been absent in CPE research. To our knowledge, it was also the first study to investigate CPE in England.

However, the study’s limitations must be considered. Our findings are limited to a small sample of seven CPE students and thus cannot be generalised to all healthcare chaplains in England. Our data were self-reported and did not incorporate observations from others, such as supervisors, colleagues, or care recipients, which are often viewed as more objective. The findings warrant careful interpretation, given that participants’ investment and belief in their growth and desire to be perceived as having improved could have contributed to overestimating their pre-post changes.

## Conclusion

CPE participants endorsed the distinct benefits and effectiveness of CPE. They reported gains in chaplain capabilities, emotional intelligence, and counselling self-efficacy and provided nuanced descriptions of their transformational learning, personal development, strengthened professional confidence, identity, and functioning, and growth in reflective practice and chaplaincy competencies serving diverse care recipients. The evaluation of this CPE unit demonstrated the effectiveness and value of the CPE model for chaplains with diverse experience levels and backgrounds in England. This study also contributed to our understanding of the development pathways and how interconnected educational factors promoted growth among chaplain participants. Findings were consistent with and expanded the body of research on CPE. Finally, we conceptualized preliminary models for chaplain development and learning pathways in CPE that need further validation and refinement by future research.

## Supporting information

S1 Checklist Inclusivity in global research. (PDF)

S1 Table Good Reporting of a Mixed Methods Study (GRAMMS) guideline. (PDF)

S2 Table Standards for Reporting Qualitative Research (SRQR) Guideline. (PDF)

S3 Table Focus group session guide and questions. (PDF)

S4 Table Changes associated with CPE participation: Pre-post survey results. (PDF)

S5 Table Net Promoter Score (NPS) results with comments reflecting CPE participant experience. (PDF)

S1 Fig Chaplain Capabilities Scale: Pre-post changes. (PDF)

## References

[1] Association of Professional Chaplains (APC). Requirements and Definitions for Board Certified & Associate Certified Chaplains. In: APC BCCI [Internet]. 2024. Available: https://www.apchaplains.org/bcci-site/becoming-certified/qualifications-for-board-certified-associate-certified-chaplains/

[2] Canadian Association for Spiritual Care / Association canadienne de soins spirituels (CASC/ACSS). Certification: Overview & Requirements. In: CASC/ACSS [Internet]. Available: https://www.spiritualcare.ca/overview—requirements.html

[3] The Healthcare Chaplaincy Board (HCB). Standards for certification in Healthcare chaplaincy 2018. The Healthcare Chaplaincy Board (HCB); 2018.

[4] Chaplaincy Accreditation Board (CAB). Certification of Healthcare Chaplains and Recognised Standards. Chaplaincy Accreditation Board (CAB); 2014.

[5] Spiritual Care Australia (SCA). Becoming a Spiritual Care Practitioner. In: Spiritual Care Australia (SCA) [Internet]. 2023. Available: https://www.spiritualcareaustralia.org.au/about-us/becoming-a-spiritual-care-practitioner/

[6] Spiritual Care Australia (SCA), Spiritual Health Association (SHA). Certification for spiritual care practitioners in health. Spiritual Care Australia (SCA);

[7] Snorton TE. Setting common standards for professional chaplains in an age of diversity. South Med J. 2006;99: 660+. doi: 10.1097/01.smj.0000222404.81215.03 16800436

[8] Caring for the Spirit: A strategy for the chaplaincy and spiritual healthcare workforce. South Yorkshire NHS Workforce Development Confederation; 2003.

[9] Zollfrank AA, Garlid CF. Part II: Clinical Pastoral Education. In: Cobb MR, Puchalski CM, Rumbold B, editors. Oxford Textbook of Spirituality in Healthcare. Oxford University Press; 2012.

[10] ACPE: The Standard for Spiritual Care & Education (ACPE). ACPE Manuals: 2020 Accreditation Manual. In: ACPE [Internet]. 2020. Available: https://www.manula.com/manuals/acpe/acpe-manuals/2016/en/topic/2020-accreditation-manual-new-process

[11] Canadian Association for Spiritual Care / Association canadienne de soins spirituels (CASC/ACSS). Educational Programs. In: CASC/ACSS [Internet]. Available: https://www.spiritualcare.ca/overview.html

[12] Australia and New Zealand Association for Clinical Pastoral Education Ltd (ANZACPE). Standards & Guidelines. In: ANZACPE [Internet]. 2023. Available: https://www.anzacpe.org.au/standards-committees/

[13] Association of Clinical Pastoral Education (Ireland) Ltd (ACPEI). Standards & Policies. ACPEI; 2017. Available: https://www.acpeireland.com/standards-and-policies

[14] Tartaglia AF. Reflection on the Development and Future of Chaplaincy Education. Reflective Practice: Formation and Supervision in Ministry. 2015. Available: https://journals.sfu.ca/rpfs/index.php/rpfs/article/view/391/382

[15] Tartaglia A, Dodd-McCue D. Enhancing objectivity in pastoral education: use of standardized patients in video simulation. J Pastoral Care Counsel. 2010;64: 2.1–10. doi: 10.1177/154230501006400202 20828071

[16] VandeCreek L, Hover M, Gleason JJ. Quantitative outcomes of Clinical Pastoral Education: A review of the literature. Journal of Supervision and Training in Ministry. 2001;21: 132–147.

[17] Fitchett G, Gray GT. Evaluating the outcome of clinical pastoral education: A test of the clinical ministry assessment profile. Journal of Supervision and Training in Ministry. 1994;15: 3–22.

[18] Jackson-Jordan E, Stafford C, Stratton SV, Vilagos TT, Janssen Keenan A, Hathaway G. Evaluation of a Chaplain Residency Program and Its Partnership with an In-Patient Palliative Care Team. J Health Care Chaplain. 2018;24: 20–29. doi: 10.1080/08854726.2017.1324088 28535117

[19] Jankowski KRB, Vanderwerker LC, Murphy KM, Montonye M, Ross AM. Change in pastoral skills, emotional intelligence, self-reflection, and social desirability across a unit of CPE. J Health Care Chaplain. 2008;15: 132–148. doi: 10.1080/08854720903163304 19994611

[20] O’Connor TJ, Fox KA, Meakes E, Empey G, O’Neill K. Quantitative and qualitative outcome research on a regional basic supervised SPE (supervised pastoral education) program. J Pastoral Care. 1997;51: 195–206. doi: 10.1177/002234099705100207 10169315

[21] Vanderstelt H, van Dijk A, Lasair S. Transformational education: exploring the lasting impact of students’ clinical pastoral education experiences. J Health Care Chaplain. 2023;29: 89–104. doi: 10.1080/08854726.2022.2040892 35189783

[22] Trothen TJ. Students’ perspectives: a Canadian study of supervised pastoral education. J Pastoral Care. 2000;54: 325–337. doi: 10.1177/002234090005400309 11146999

[23] Fetters MD, Curry LA, Creswell JW. Achieving integration in mixed methods designs-principles and practices. Health Serv Res. 2013;48: 2134–2156. doi: 10.1111/1475-6773.12117 24279835 PMC4097839

[24] O’Cathain A, Murphy E, Nicholl J. The quality of mixed methods studies in health services research. J Health Serv Res Policy. 2008;13: 92–98. doi: 10.1258/jhsrp.2007.007074 18416914

[25] O’Brien BC, Harris IB, Beckman TJ, Reed DA, Cook DA. Standards for reporting qualitative research: a synthesis of recommendations. Acad Med. 2014;89: 1245–1251. doi: 10.1097/ACM.0000000000000388 24979285

[26] Schutte NS, Malouff JM, Hall LE, Haggerty DJ, Cooper JT, Golden CJ, et al. Development and validation of a measure of emotional intelligence. Pers Individ Dif. 1998;25: 167–177. doi: 10.1016/S0191-8869(98)00001-4

[27] Schutte NS, Malouff JM, Bhullar N. The Assessing Emotions Scale. In: Parker JDA, Saklofske DH, Stough C, editors. Assessing Emotional Intelligence: Theory, Research, and Applications. Boston, MA: Springer US; 2009. pp. 119–134. doi: 10.1007/978-0-387-88370-0_7

[28] Lent RW, Hill CE, Hoffman MA. Development and validation of the Counselor Activity Self-Efficacy Scales. J Couns Psychol. 2003;50: 97–108. doi: 10.1037/0022-0167.50.1.97

[29] Health Research Authority (HRA). Do I need NHS Ethics approval? In: NHS Health Research Authority (HRA) Decision Tool. [Internet]. Available: https://www.hra-decisiontools.org.uk/ethics/

[30] Health Research Authority (HRA). Does my project require review by a Research Ethics Committee? V2.0. NHS Health Research Authority (HRA); 2020. Available: https://www.hra-decisiontools.org.uk/ethics/docs/Algorithm%20-%20Does%20my%20project%20require%20REC%20review%20v2.0%2020200304.pdf

[31] UK Board of Healthcare Chaplaincy (UKBHC). Spiritual and Religious Care Capabilities and Competences for Healthcare Chaplains. UKBHC; 2015. Available: https://www.ukbhc.org.uk/for-employers/standards-competencies/

[32] Braun V, Clarke V. Using thematic analysis in psychology. Qual Res Psychol. 2006;3: 77–101. doi: 10.1191/1478088706qp063oa

[33] Braun V, Clarke V. Thematic analysis: A practical guide. SAGE; 2021.

[34] Braun V, Clarke V. Conceptual and design thinking for thematic analysis. Qualitative Psychology. 2022;9: 3–26. doi: 10.1037/qup0000196

[35] Mayer JD, Salovey P. What is emotional intelligence? In: Sluyter DJ, editor. Emotional development and emotional intelligence: Educational implications. New York, NY: Basic Books; 1997. pp. 3–34.

[36] Trothen TJ. Canadian supervised pastoral education—affirmations and ethical queries emerging from a two-year study. J Pastoral Care. 2001;55: 365–377. doi: 10.1177/002234090105500403 11799644

[37] Hunsmann JJ, Ay-Bryson DS, Kobs S, Behrend N, Weck F, Knigge M, et al. Basic counseling skills in psychology and teaching: validation of a short version of the counselor activity self-efficacy scales. BMC Psychol. 2024;12: 32. doi: 10.1186/s40359-023-01506-7 38238872 PMC10797791

[38] Gockel A, Burton DL. An Evaluation of Prepracticum Helping Skills Training for Graduate Social Work Students. J Soc Work Educ. 2014;50: 101–119. doi: 10.1080/10437797.2014.856234

[39] den Toom NJ. The Chaplain-Researcher: The Perceived Impact of Participation in a Dutch Research Project on Chaplains’ Professionalism. PhD, Protestantse Theologische Universiteit. 2022. Available: https://research.tilburguniversity.edu/files/65715947/Den_Toom_2022_The_Chaplain_Researcher_met_omslag.pdf

[40] den Toom N, Visser A, Körver J, Walton MN. The perceived impact of being a chaplain-researcher on professional practice. J Health Care Chaplain. 2024; 1–14. doi: 10.1080/08854726.2022.2132036 36264014

[41] Szilagyi C, Vandenhoeck A, Best MC, Desjardins CM, Drummond DA, Fitchett G, et al. Chaplain Leadership During COVID-19: An International Expert Panel. J Pastoral Care Counsel. 2022;76: 56–65. doi: 10.1177/15423050211067724 34931932 PMC8926913

[42] Szilagyi C, Tartaglia A, Palmer PK, Fleenor DW, Jackson-Jordan E, Knoll Sweeney S, et al. COVID-19 and Clinical Pastoral Education: How ACPE Educators Pivoted Amid the Pandemic. J Pastoral Care Counsel. 2022;76: 37–47. doi: 10.1177/15423050211073572 35060791 PMC8926918

[43] Szilagyi C, Tartaglia A, Palmer PK, Fleenor DW, Jackson-Jordan E, Knoll Sweeney S, et al. Delivering Clinical Pastoral Education (CPE) Remotely: Educators’ Views and Perspectives During the COVID-19 Pandemic and Beyond. J Pastoral Care Counsel. 2022;76: 189–209. doi: 10.1177/15423050221094492 35499920 PMC9066239

[44] Kestenbaum A, Winters KD, Ruppin-Pham A, Valdez MJ, Cammon C, Hamelin K, et al. Improving access to palliative care clinical pastoral education. J Health Care Chaplain. 2023;29: 320–335. doi: 10.1080/08854726.2023.2209464 37184137

