# Supplementary material for: Chaplain development in Clinical Pastoral Education (CPE) in healthcare settings in England: A mixed methods study
Source: PLoS One. 2024 Sep 11;19(9):e0310085. doi: 10.1371/journal.pone.0310085 (PMC11389922; doi:10.1371/journal.pone.0310085)
Supplement: S1 Table — (PDF) [file pone.0310085.s002.pdf]

**S1 Table. Good Reporting of a Mixed Methods Study (GRAMMS) Guideline**

| **Good Reporting of a Mixed Methods Study (GRAMMS) Guideline** (O'Cathain et al., 2008) | **Section: Page** |
|---|---|
| Describe the justification for using a mixed methods approach to the research question | Methods: 5-6 |
| Describe the design in terms of the purpose, priority and sequence of methods | Methods: 5-6 |
| Describe each method in terms of sampling, data collection and analysis | Methods: 6-10 |
| Describe where integration has occurred, how it has occurred and who has participated in it | Methods: 5-6<br>Discussion: 34-35 |
| Describe any limitation of one method associated with the present of the other method | Discussion: 35, 39 |
| Describe any insights gained from mixing or integrating methods | Discussion: 34-35, 39 |

**Reference**

O'Cathain, A., Murphy, E., & Nicholl, J. (2008). The quality of mixed methods studies in health services research. *Journal of Health Services Research & Policy*, *13*(2), 92–98. https://doi.org/10.1258/jhsrp.2007.007074
