# Supplementary material for: Chaplain development in Clinical Pastoral Education (CPE) in healthcare settings in England: A mixed methods study
Source: PLoS One. 2024 Sep 11;19(9):e0310085. doi: 10.1371/journal.pone.0310085 (PMC11389922; doi:10.1371/journal.pone.0310085)
Supplement: S3 Table — (PDF) [file pone.0310085.s004.pdf]

S4 Table. Focus Group Session Guide and Questions

Questions	Notes and Potential Probing Questions
**Learning and Growth in CPE**	
1. What was most useful in the CPE learning process?	Can you give an example of what you're describing?
2. What was most challenging for you in CPE?	
**Learning about Self**	
3. What was the most important thing you learned about yourself in CPE?	Either as a person or as a chaplain.
**Interpersonal and Clinical Skills**	
4. What was the most important thing you learned about how you relate to others in CPE?	Can you give us an example of how your interactions or relationships with others (e.g., patients or colleagues) have changed?
5. How did CPE impact or change your spiritual care with patients and others (e.g., families, staff, etc.)?	Can you give an example from your care with patients or colleagues?
6. How did CPE influence or change your ability to listen to persons' deeper story?	What exactly changed? Examples?
**Chaplaincy Capabilities**	
7. What chaplaincy skill/capability did you improve the most in CPE?	How so? Can you give an example of how your spiritual care with patients or care recipients has changed?
8. How did CPE influence your confidence in providing care to persons outside your particular faith group?	Can you give us an example of serving someone from a different faith group more effectively because of your CPE learning? What was different in your approach because of CPE?
9. How did CPE impact your capacity to effectively engage in highly intense spiritual care situations?	
10. How has CPE contributed to your capacity as a reflective practitioner?	
11. How did CPE help you develop in your chaplain role or identity? How did CPE shift your view of yourself as a professional chaplain?	Do you look at yourself as a chaplain differently? How did CPE impact your professionalism as a chaplain?
12. How did your sense of functioning as a chaplain change within your chaplaincy teams?	Can you give us an example of how your interactions and functioning with other chaplains have changed?
13. How did CPE impact your ability to claim your sense of professionalism in interdisciplinary teams?	Can you give us an example regarding your interactions and professionalism as a member of interdisciplinary teams?
14. How did this CPE unit impact your awareness of or confidence in spiritual care assessment and planning?	
15. How did CPE develop your understanding of leadership or your ability to take the lead in your chaplain role?	For example, taking the lead in various clinical situations, how you represent your profession, or taking up your voice and influence beyond your role as a healthcare professional?

**Utility of CPE for Chaplaincy Training**	
16. How did CPE compare to your prior chaplaincy training/education/preparation?	
17. How well would CPE prepare others for chaplaincy and spiritual care?	
18. At what point of chaplaincy training/preparation would CPE be the most useful?	
18/B. If not answered in question 18 or above:<br>How do you feel CPE would benefit someone entering into chaplaincy?	
19. How did you find the intensity of taking CPE over 4 months?	How would it be for someone entering chaplaincy?
20. In terms of the structure of the course, is there anything you would change or tweak?	
