# Supplementary material for: Chaplain development in Clinical Pastoral Education (CPE) in healthcare settings in England: A mixed methods study
Source: PLoS One. 2024 Sep 11;19(9):e0310085. doi: 10.1371/journal.pone.0310085 (PMC11389922; doi:10.1371/journal.pone.0310085)
Supplement: S4 Table — (PDF) [file pone.0310085.s005.pdf]

S5 Table. Changes Associated with CPE Participation: Pre-Post Survey Results

Variable	Pre-CPE (mean)	Post-CPE (mean)	Pre-Post % change
**Chaplaincy Capabilities Scale**			
Chaplaincy Capabilities Scale [a]	3.7	4.2	12%
**Emotional Intelligence (SREIT)**			
Emotional Intelligence (SREIT total) [b]	125.3	134.0	7%
Emotional Intelligence (SREIT average)	3.8	4.1	7%
Perception of Emotion [a]	3.9	4.2	6%
Managing Own Emotions [a]	3.6	4.0	11%
Managing Others' Emotions [a]	3.8	4.0	6%
Utilization of Emotions [a]	3.9	4.1	6%
**Counselling Self-Efficacy (CASES)**			
Exploration Skills Self-Efficacy [c]	7.3	7.6	4%
Insight Skills Self-Efficacy [c]	5.9	6.6	12%
Action Skills Self-Efficacy [c]	5.3	5.9	13%
Session Management Self-Efficacy [c]	6.4	7.3	15%
**Chaplain Capabilities Scale**			
Chaplaincy Capabilities Scale [a]	3.7	4.2	12%

Note: [a] Instrument scale: 1-5; [b] SREIT instrument total score range: 33-165; [c] CASES instrument scale: 0-9; SREIT: Self-Report Emotional Intelligence Test (SREIT). For overall emotional intelligence and four subscales, scores were also calculated by using mean scores to allow comparison of scales with varying numbers of items (Schutte et al., 1998; Schutte et al., 2009).; CASES: Counselor Activity Self-Efficacy Scales (CASES) Part 1 and Part 2 (Lent et al., 2003).

References

Lent, R. W., Hill, C. E., & Hoffman, M. A. (2003). Development and validation of the Counselor Activity Self-Efficacy Scales. *Journal of Counseling Psychology*, *50*(1), 97–108. https://doi.org/10.1037/0022-0167.50.1.97

Schutte, N. S., Malouff, J. M., & Bhullar, N. (2009). The Assessing Emotions Scale. In J. D. A. Parker, D. H. Saklofske, & C. Stough (Eds.), *Assessing Emotional Intelligence: Theory, Research, and Applications* (pp. 119–134). Springer US. https://doi.org/10.1007/978-0-387-88370-0_7

Schutte, N. S., Malouff, J. M., Hall, L. E., Haggerty, D. J., Cooper, J. T., Golden, C. J., & Dornheim, L. (1998). Development and validation of a measure of emotional intelligence. *Personality and Individual Differences*, *25*(2), 167–177. https://doi.org/10.1016/S0191-8869(98)00001-4
