# Supplementary material for: Chaplain development in Clinical Pastoral Education (CPE) in healthcare settings in England: A mixed methods study
Source: PLoS One. 2024 Sep 11;19(9):e0310085. doi: 10.1371/journal.pone.0310085 (PMC11389922; doi:10.1371/journal.pone.0310085)
Supplement: S5 Table — (PDF) [file pone.0310085.s006.pdf]

S7 Table. Net Promoter Score (NPS) Results with Comments Reflecting CPE Participant Experience (post-CPE)

**Net Promoter Score (NPS)** [a]	**100**
Promoters	100%
Passives	0%
Detractors	0%
Median score (IQR); range	10 (0.0); 9-10
**What is the primary reason for your rating?** (Free-Text Responses)	
"My experience of CPE has been one of both personal and professional learning. It has been transformational in terms of my self awareness and professional development as a Healthcare Chaplain."	
"An experiential, academically-robust, transformational course which integrates the chaplain's personal, pastoral and professional identity, developing their chaplaincy skills and knowledge in order to work to the best of their potential in a health care setting."	
"I think all chaplains need to be able to understand and value themselves, CPE teaches you this to enable them to give congruent and genuine support."	
"Fantastic way of learning a huge amount in a short space of time. It was brilliant to grow personally as well as professionally."	
"I believe in the importance of the chaplain's self awareness as a living document to be able to appropriately provide support to the service users. Combined with Theoretical foundation and praxis."	
"It has increased my self awareness and my freedom in ministry. Each group member can set their own learning goals and so have a unique learning path. The action-reflection-action model focuses on what needs to be changed in ministry."	
"CPE has really nurtured me in my thought process, my practice and how I am presenting myself when ministering to those around me. It has helped me develop my self awareness and empowered me to challenge myself and to believe in myself as a chaplain and recognise the qualities and gifts I bring as a chaplain but to also explore how I need to develop further."	

Note: [a] NPS is calculated by subtracting the percentage of detractors (response range 0-6) from the percentage of promoters (response range 9-10).
