# Supplementary material for: Chaplain development in Clinical Pastoral Education (CPE) in healthcare settings in England: A mixed methods study
Source: PLoS One. 2024 Sep 11;19(9):e0310085. doi: 10.1371/journal.pone.0310085 (PMC11389922; doi:10.1371/journal.pone.0310085)
Supplement: S1 Fig — (PDF) [file pone.0310085.s007.pdf]

S6 Fig. Chaplain Capabilities Scale: Pre-Post Changes

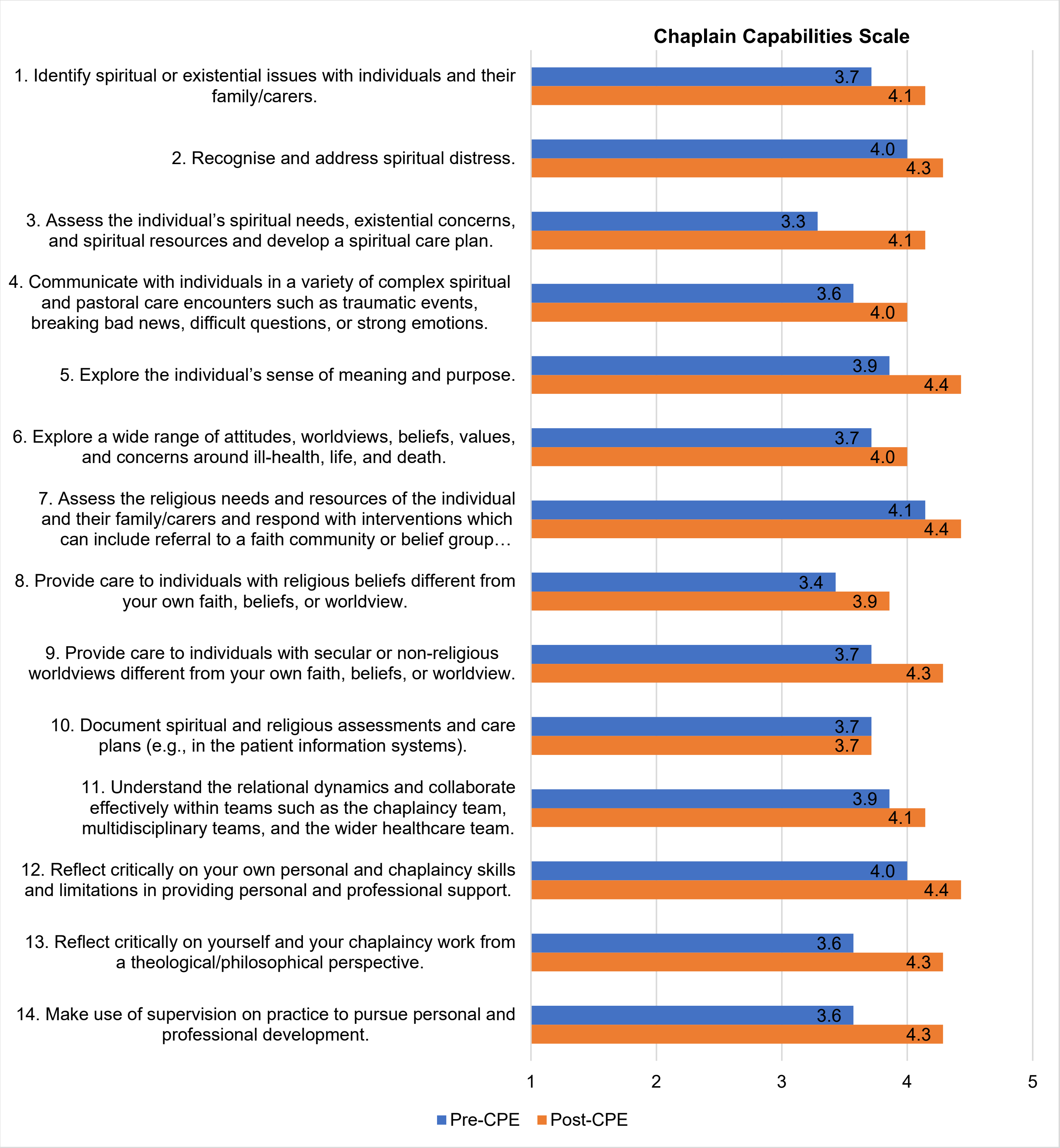

S5 Figure presents Chaplain Capabilities Scale (CCS) mean scores. The 14-item CCS was developed by the authors and was informed by the Spiritual and Religious Care Capabilities and Competences for Healthcare Chaplains by the UK Board of Healthcare Chaplaincy (UKBHC, 2015) and the ACPEI CPE Learning Outcomes (ACPEI, 2017).

References

Association of Clinical Pastoral Education (Ireland) Ltd (ACPEI). (2017). *Standards & Policies*. ACPEI. https://www.acpeireland.com/standards-and-policies

UK Board of Healthcare Chaplaincy (UKBHC). (2015). *Spiritual and Religious Care Capabilities and Competences for Healthcare Chaplains*. UKBHC. https://www.ukbhc.org.uk/for-employers/standards-competencies/
